# Programmable Bispecific Nano-immunoengager That Captures T Cells and Reprograms Tumor Microenvironment

**DOI:** 10.1021/acs.nanolett.2c00582

**Published:** 2022-08-04

**Authors:** Lu Zhang, Ruonan Bo, Yi Wu, Longmeng Li, Zheng Zhu, Ai-Hong Ma, Wenwu Xiao, Yanyu Huang, Tatu Rojalin, Xingbin Yin, Chunping Mao, Fengyi Wang, Yongheng Wang, Hongyong Zhang, Kelmen E. Low, Kiana Lee, Yousif Ajena, Di Jing, Dalin Zhang, Christopher M. Baehr, Ruiwu Liu, Lei Wang, Yuanpei Li, Kit S. Lam

**Affiliations:** † Department of Biochemistry and Molecular Medicine, UC Davis NCI-designated Comprehensive Cancer Center, University of California Davis, Sacramento, California 95817, United States; ‡ Department of Biomedical Engineering, Southern University of Science and Technology, Shenzhen, Guangdong 518055, China; § CAS Center for Excellence in Nanoscience, CAS Key Laboratory for Biomedical Effects of Nanomaterials and Nanosafety, National Center for Nanoscience and Technology, Beijing 100190, China; ∥ Division of Hematology and Oncology, Department of Internal Medicine, School of Medicine, University of California Davis, Sacramento, California 95817, United States

**Keywords:** nano-immuno-engager, fibrillar transformation, T cells capture, immune checkpoint blockade (ICB) therapy

## Abstract

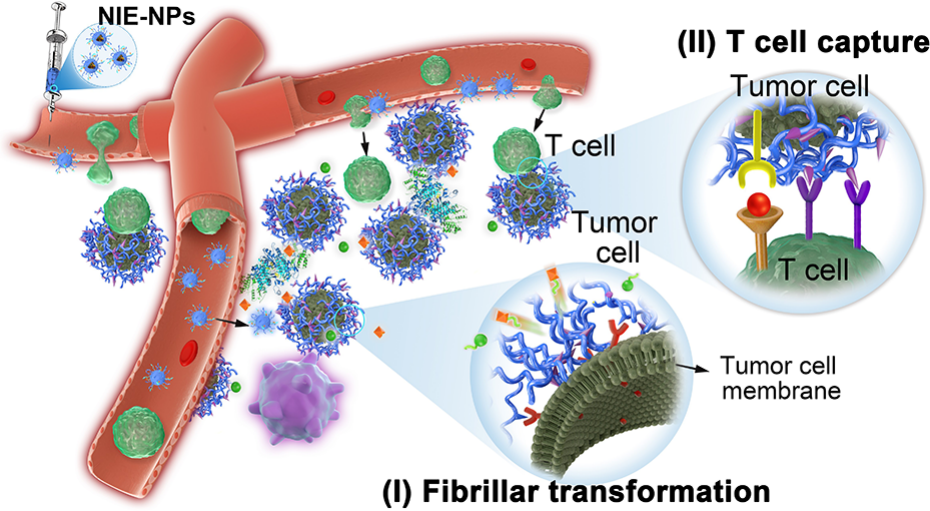

Immune checkpoint blockade (ICB) therapy has revolutionized clinical oncology. However, the efficacy of ICB therapy is limited by the ineffective infiltration of T effector (T_eff_) cells to tumors and the immunosuppressive tumor microenvironment (TME). Here, we report a programmable tumor cells/T_eff_ cells bispecific nano-immunoengager (NIE) that can circumvent these limitations to improve ICB therapy. The peptidic nanoparticles (NIE-NPs) bind tumor cell surface α_3_β_1_ integrin and undergo *in situ* transformation into nanofibrillar network nanofibers (NIE-NFs). The prolonged retained nanofibrillar network at the TME captures T_eff_ cells via the activatable α_4_β_1_ integrin ligand and allows sustained release of resiquimod for immunomodulation. This bispecific NIE eliminates syngeneic 4T1 breast cancer and Lewis lung cancer models in mice, when given together with anti-PD-1 antibody. The *in vivo* structural transformation-based supramolecular bispecific NIE represents an innovative class of programmable receptor-mediated targeted immunotherapeutics to greatly enhance ICB therapy against cancers.

Immune checkpoint receptor pathway blockade monoclonal antibodies such as anti-PD-1, anti-PD-L1, and anti-CTLA-4 can reverse T effector (T_eff_) cell dysfunction and exhaustion, resulting in dramatic tumor shrinkage and sometimes complete remission in some patients, even with late stage metastatic diseases.^1,2^ However, the response rate varies greatly between tumor types: up to 40% in melanoma, 25% in non-small cell lung cancer, but <10% in most other tumor types. The low response rate of immune checkpoint blockade (ICB) therapy is probably ascribed to defects in T_eff_ cell infiltration at the tumor sites.^3^ In addition, the tumor microenvironment (TME) can also influence tumor response to ICB therapies.^4^

Advancement and optimization of nano-immunotherapy lie in the development of innovative approaches to enhance the specificity and controllability of immunotherapeutic interventions and in the targeting of desired cell types. Advanced bionanomaterials or approaches in a more controlled manner could enhance immunotherapeutic potency by increasing the accumulation and prolonging the retention of the immunomodulatory agent and capturing of the immune cells at the TME while sparing the normal tissues and organs, thus reducing off-target adverse effects such as the systemic cytokine storm.^5−10^ Especially, *in situ* modulation of nanomaterial *in vivo* has been demonstrated to improve the performance of bioactive molecules.^11−16^

In order to overcome ICB resistance, here we report on a programmable tumor cells/T_eff_ cells bispecific nano-immunoengager (NIE) that can capture T_eff_ cells at tumor sites and sustainably release immunoagonist to reprogram the TME. This bispecific NIE, initially in nanoparticle form (NIE-NPs), is self-assembled from two transformable peptide monomers (TPMs, Figure 1a). TPM1, LXY30-KLVFFK(*Pa*), was comprised of three discrete functional domains: (1) the high-affinity and high-specificity LXY30 cyclic peptide (cdG-Phe(3,5-diF)-G-Hyp-NcR) ligand that targets the α_3_β_1_ integrin heterodimeric transmembrane receptor expressed by many epithelial tumors with high metastatic potential, including clinical non-small cell lung cancer;^17−19^ (2) the KLVFF β-sheet forming peptide domain originated from β-amyloid (Aβ) peptide;^11,20−22^ and (3) the pheophorbide a (*Pa*) moiety with fluorescence property, serving as a hydrophobic core to induce the formation of micellar nanoparticles. TPM2, *pro*LLP2A-KLVFFK(*R848*), was also comprised of three discrete functional domains: (1) *pro*LLP2A, the “pro-ligand” version of LLP2A ligand that is a high-affinity and high-specificity peptidomimetic ligand against the activated α_4_β_1_ integrin of lymphocytes;^23^ (2) the same KLVFF β-sheet forming peptide domain; and (3) R848 (resiquimod), a hydrophobic TLRs 7/8 agonist, grafted to the TPM2 main chain via an ester bond. In *pro*LLP2A, the carboxyl group of LLP2A is blocked by 3-methoxy-1-propanol through esterification such that it will not interact with normal lymphocytes and mesenchymal stem cells during blood circulation.

Under aqueous conditions and in blood circulation, TPM1 and TPM2, at a ratio of 1:1, would co-self-assemble into nanoparticles, NIE-NPs, in which KLVFFK(*Pa*) and KLVFFK(*R848*) hydrophobic domains were in the interior of the NIE-NPs, while relatively hydrophilic LXY30 and *pro*LLP2A ligand peptides were on the surface of the NIE-NPs. NIE-NPs would preferentially accumulate in tumors through the leaky tumor vasculatures. Upon interaction with α_3_β_1_ integrin receptor protein displayed on the tumor cell membrane, the NIE-NPs would undergo *in situ* transformation into nanofibers (NIE-NFs) forming a nanofibrillar structural network on the surface of tumor cells, thus maintaining a prolonged retention (at least 7 days). With the abundant esterase in the TME and on the tumor cells, *pro*LLP2A would quickly be converted to LLP2A against activated α_4_β_1_ integrin. LLP2A displayed on the fibrils would capture T_eff_ cells (e.g., CD8^+^ T cells) and facilitate their long-term retention at the TME and adjacent to the tumor cells. The capturing and retention of large numbers of T_eff_ cells would greatly enhance ICB therapy.^10,24−26^ Besides, the sustained release of R848 from the nanofibrillar network would improve the immunosuppressive tumor microenvironment, e.g., activate antigen-presenting cells, promote immune cells to produce antitumor response factors, and re-educate the phenotype of the macrophage from M2 to M1.^27^ This *in vivo* structural transformation-based supramolecular bispecific NIE represents an innovative class of programmable receptor-mediated targeted immunotherapeutics against cancers through capturing of T cells and enhancement of the antitumor immune state at the TME (Scheme 1).

**Scheme 1:**
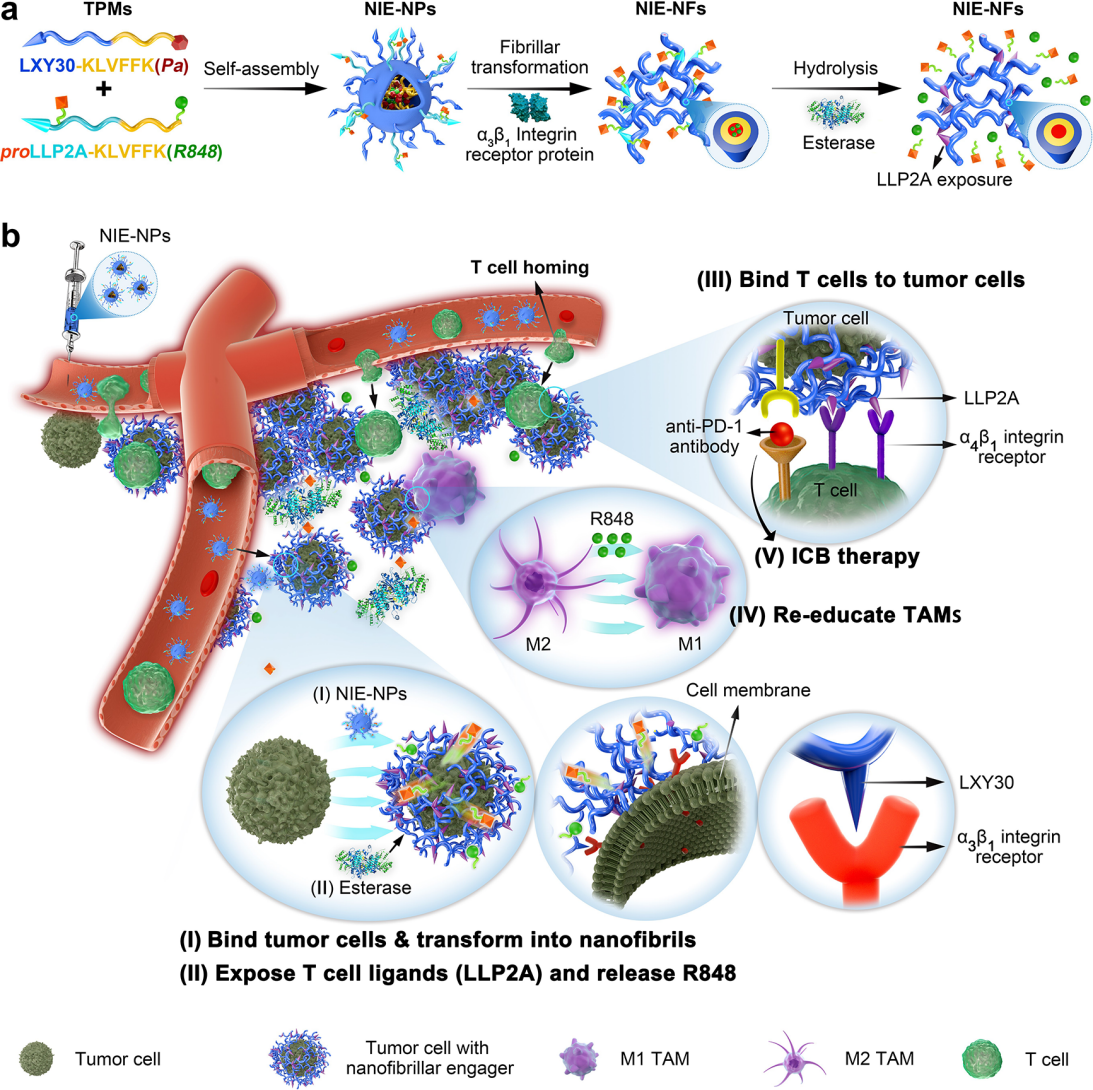
Programmable Bispecific Nano-immunoengager (NIE) Synergizing Immune Checkpoint Blockage (ICB) Therapy *via* Capturing T_eff_ Cells and Reprogramming the Tumor Microenvironment (a and b) Schematic illustration of (a) co-self-assembly of both TPM1 and TPM2 into NIE-NPs, *in situ* fibrillar transformation into NIE-NFs through binding α_3_β_1_ integrin (tumor cell membrane), exposure of LLP2A ligands binding α_4_β_1_ integrin (T cell membrane), and release of TLRs 7/8 agonist (R848) from NIE-NFs through esterase hydrolysis. (b) Steps II–V show the processes for how a programmable bispecific NIE synergizes ICB therapy in tumor tissue: (I) NIE-NPs bind tumor cells and *in situ* transforms them into nanofibrils (NIE-NFs) on the surface of tumor cells. (II) NIE-NFs expose LLP2A and release R848 for (III) capturing T cells to tumors cells and (IV) re-educating TAMs from M2 to M1 phenotype. (V) Meanwhile, anti-PD-1 antibody greatly activates the NIE homed cytotoxic T cells for ICB therapy. (TPMs, transformable peptide monomers; NIE-NPs, nano-immuno-engager nanoparticles; NIE-NFs, nano-immuno-engager nanofibrils; R848, resiquimod, a TLRs 7/8 agonist; M1-TAM, M1-like tumor-associated macrophage; M2-TAM, M2-like tumor-associated macrophage.)

TPM1 and TPM2 were synthesized and characterized (Figures 1a and S1). As the proportion of water in the mixed solvent (water and DMSO) of the TPM1 and TPM2 mixture solution was increased, there was a gradual decrease in the fluorescence peak at 675 nm due to the aggregation-caused quenching properties of *Pa* dye (Figure 1b), reflecting the gradual formation of NIE-NPs via self-assembly. Concomitantly, there was a modest decrease in the absorption peak at both 405 and 680 nm (Figure S2a). TPM1 and TPM2 each alone were able to self-assemble to form nanoparticles (NPs_TPM1_ and NPs_TPM2_) at 18 and 55 nm. NIE-NPs, assembled from a 1:1 mix of TPM1 and TPM2 into nanoparticles at around 28 nm, which fell between the sizes of NPs_TPM1_ and NPs_TPM2_ (Figure S2b). The critical aggregation concentration (CAC) of the NIE-NPs was determined to be 8 μM (Figure S2c). We have also demonstrated that NIE-NPs could maintain good serum stability and proteolytic stability over 7 days at 37 °C (Figure S2d).

**Figure 1:**
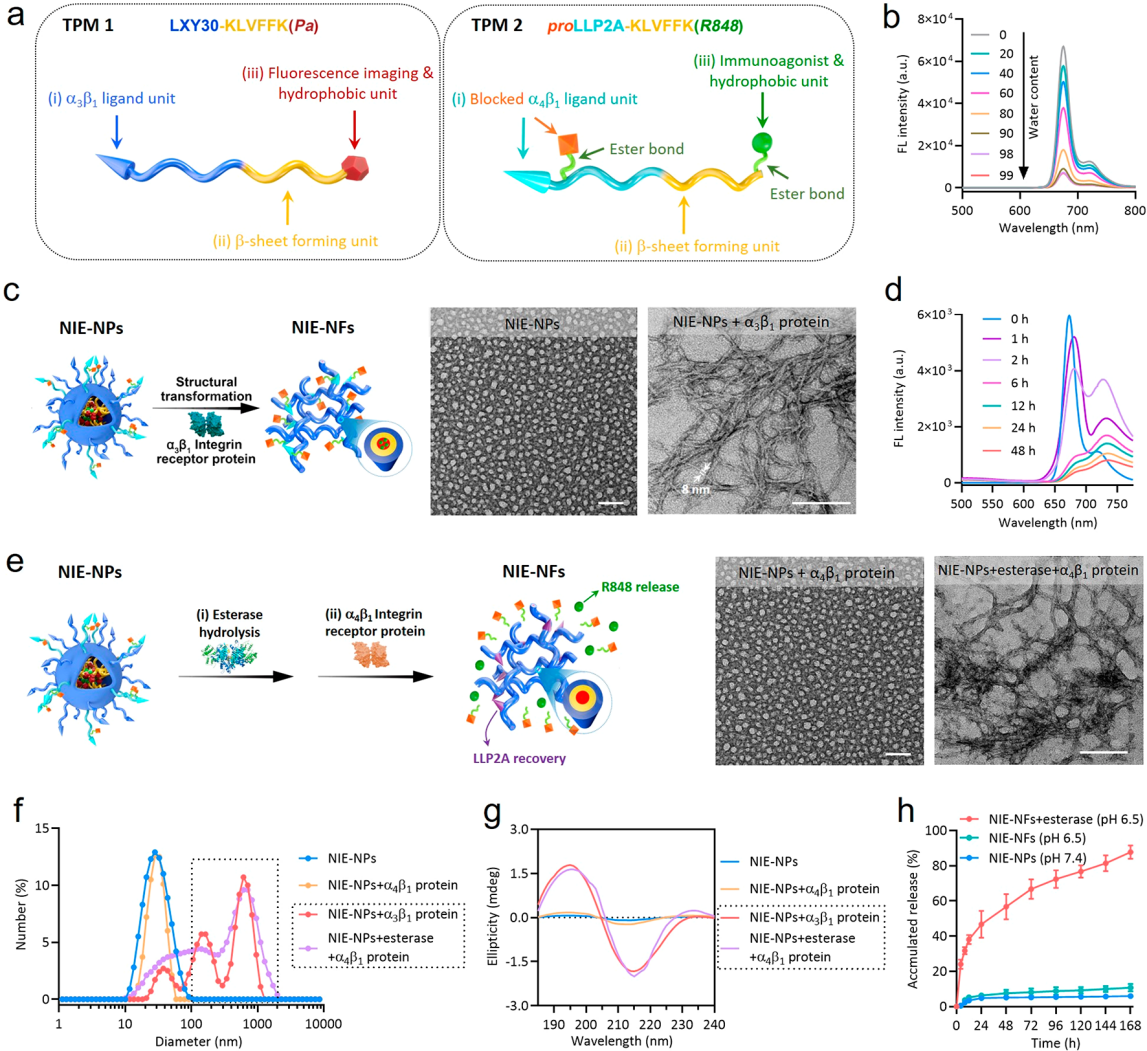
Assembly and fibrillar transformation of the programmable bispecific NIE, as well as esterase-induced exposure of LLP2A ligands and R848 release. (a) Schematic illustration of the molecular structure and function of TPM1 (LXY30-KLVFFK(*Pa*)) and TPM2 (*pro*LLP2A-KLVFFK(*R848*)). (b) Changes in fluorescence (FL) of a DMSO solution of TPM1 and TPM2 at a 1:1 ratio following the gradual addition of water (from 0 to 99%) forming micellar NIE-NPs: excitation wavelength, 405 nm. (c) Schematic illustration and TEM images of initial NIE-NPs and subsequently transformed nanofibrils (NIE-NFs) upon interaction with soluble α_3_β_1_ integrin protein for 24 h (H_2_O to DMSO ratio of 99:1). The concentration of NIE-NPs used in the experiment was 20 μM. Scale bars are 100 nm. (d) Variation in the fluorescence (FL) signal of *Pa* in the fibrillar-transformation process of NIE-NPs to NIE-NFs over time. (e) Schematic illustration and TEM images of NIE-NPs upon interaction with soluble α_4_β_1_ integrin protein or α_4_β_1_ integrin protein plus esterase for 24 h (H_2_O to DMSO ratio of 99:1), respectively. The concentration of NIE-NPs used in the experiment was 20 μM. Scale bars are 100 nm. (f and g) Variation in the size distribution (f) and circular dichroism spectra (g) of NIE-NPs and NIE-NFs under different conditions. (h) *In vitro* release profile of R848 from NIE-NFs over time under different conditions. Data are presented as mean ± s.d., *n* = 3 independent experiments. The molar ratio of α_3_β_1_ or α_4_β_1_ integrin protein to peptide ligand was approximately 1:1000. a.u., arbitrary units; mdeg, millidegrees.

To verify the receptor-mediated fibrillar transformable process of NIE-NPs *in vitro*, soluble α_3_β_1_ integrin protein (receptor for LXY30) was added to the solution of NIE-NPs. After 24 h of incubation at room temperature, a fibrillar network (NIE-NFs, width diameter about 8 nm) with a broad size distribution was clearly detected (Figure 1c and f). No transformation was observed in the preparation of NIE-NPs without the addition of α_3_β_1_ integrin protein, even after 24 h (Figure S2e). Addition of α_3_β_1_ integrin protein triggered a gradual decrease in the fluorescence intensity of *Pa* over time, reflecting an increase in fluorescence quenching in NFs1, indicating that the packing of *Pa* in NIE-NFs was denser (Figure 1d). The CAC of NIE-NFs was confirmed to be lower than that of NIE-NPs (5 μM vs 8 μM, Figure S2f). In addition, a fluorescence peak reversal (from 680 to 725 nm) was observed, indicating that the disordered arrangement of *Pa* in the core of NIE-NPs was transformed into *J*-aggregate form in NIE-NFs.^28−30^ We also investigated the responsiveness of *pro*LLP2A displayed on the NIE-NPs surface to soluble α_4_β_1_ integrin protein in the presence and absence of esterase (Figure 1e and f and Figure S3). Successive addition of esterase, followed by soluble α_4_β_1_ integrin protein, was able to elicit conversion of NIE-NPs to fibrillar network after 24 h of incubation. This expected result confirmed that esterase was able to convert pro-ligand *pro*LLP2A to ligand LLP2A, which in turn was able to trigger receptor-mediated transformation of NIE-NPs to NIE-NFs. We monitored the conversion of *pro*LLP2A ligand to LLP2A ligand by HPLC. The majority of *pro*LLP2A was found to be converted to LLP2A after incubation with esterase for 8 h at pH 7.4 and 37 °C (Figure S4).

Circular dichroism (CD) spectroscopic analysis of the transformation process of NIE-NPs showed a gradual progression of a negative signal at 216 nm and a positive signal at 195 nm upon incubation with α_3_β_1_ integrin protein or combination esterase/α_4_β_1_ integrin protein, indicative of β-sheet formation (Figure 1g).^20,31^ The *in vitro* release behavior of R848 from NIE-NFs was studied at pH 6.5 with addition of esterase to simulate TME conditions. About 45% of R848 was released in the first 24 h, after which the release rate gradually slowed down and about 86% cumulative release was observed by 168 h, indicating that prolonged and sustained release of R848 could occur at the TME (Figure 1h). To demonstrate the unique transformable property of NIE-NPs, we designed a related negative control nano-immunoengager nanoparticle (CNIE-NP) formed by assembly of two negative control TPMs without a β-sheet forming KLVFF peptide sequence, at a ratio of 1:1 (CTPM3:LXY30-KAAGGK(*Pa*) and CTPM4:*pro*LLP2A-KAAGGK(*R848*), Figures S1 and S5).

We have previously reported the binding affinity of LLP2A against α_4_β_1_ integrin to be at the sub-nanomolar level^23^ and that of LXY30 against α_3_β_1_ integrin to be at the mid-nanomolar level.^17^ LXY30 bound strongly to α_3_β_1_ integrin but not α_4_β_1_ integrin, and the reverse was true for LLP2A (Figure S6a and c). Esterification of the carboxyl side chain of LLP2A to form *pro*LLP2A resulted in complete loss of its binding to α_4_β_1_ integrin. Treatment of *pro*LLP2A with esterase was able to completely restore the binding affinity of LLP2A toward α_4_β_1_ integrin (Figure S6b). We have also confirmed the high binding affinity and selectivity of LXY30 to α_3_β_1_ integrin on the surface of 4T1 cells via flow cytometry (Figure S7) and also found that NIE-NPs were slightly cytotoxic against 4T1 tumor cells, with 84.5% cell viability at 50 μM (Figure S8). We investigated the distribution of nanoparticles by tracking the red fluorescent signal emitted by *Pa* using confocal laser scanning microscopy (CLSM). Six hours after incubation of 4T1 tumor cells with NIE-NPs, a strong red fluorescence signal was observed on the cell surface but not inside the cells (Figure 2a). The red fluorescence signal of transformed NIE-NPs was found to merge with plasma membrane on the cell surface but not with intracellular actin (Figures S9 and S10). In contrast, the fluorescent signal of *Pa* in the CNIE-NPs-treated group was found to be concentrated primarily in the cytoplasm of the cells. To study the retention and stability of the formed nanofibrillar network on the surface of tumor cells, unbound nanoparticles were washed off after 6 h of incubation and fresh medium without nanoparticles was added to incubate cells for another 18 h. As expected, NIE-NPs-treated cells still retained strong red fluorescence signals on the cell surface at 24 h, displaying the capacity of prolonged retention of the bispecific NIE (Figure 2b). In sharp contrast, only a weak fluorescence signal was observed inside the cells treated with CNIE-NPs after 24 h. SEM images confirmed the presence of a nanofibrillar network on the surface of 4T1 tumor cells (Figure 2c). We also investigated the effect of esterase on the interactions between NIE-NPs and CD8 T cell surface α_4_β_1_ integrin (preactivated by Mn^2+^), after converting *pro*-LLP2A to LLP2A (Figure 2d). SEM confirmed the presence of a fibrillar network on the surface of esterase pretreated NIE-NPs-treated CD8 T cells (Figure 2e).

**Figure 2:**
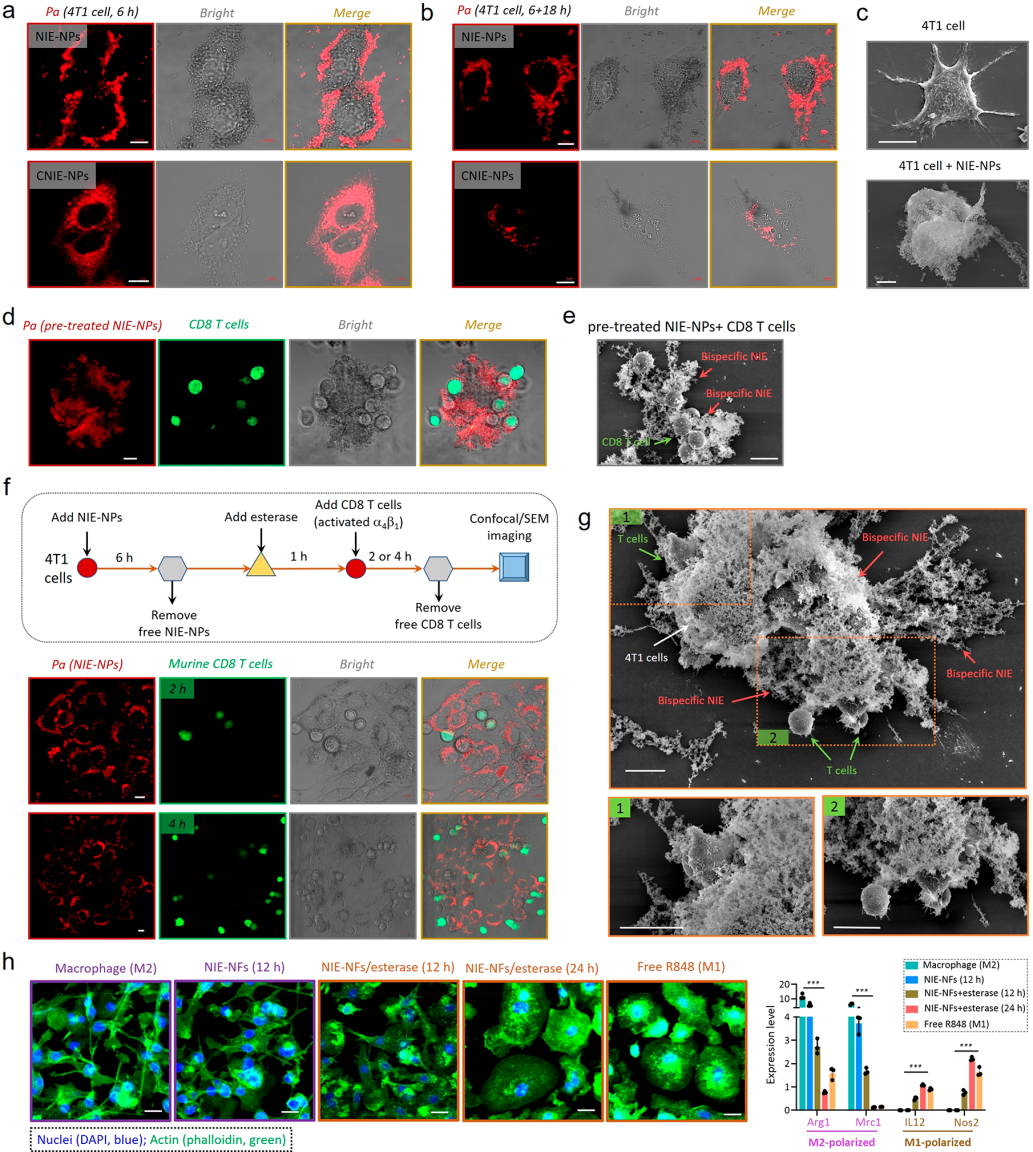
*In vitro* bispecific NIE binds both 4T1 breast cancer cells and CD8 T cells and re-educates tumor-associated macrophages. (a) Cellular fluorescence distribution images of interaction of NIE-NPs and CNIE-NPs for 6 h with 4T1 tumor cells to show NIE-NPs around cells and CNIE-NPs inside cells. Scale bar is 10 μm. (b) Cellular fluorescence signal retention images of 4T1 tumor cells after exposure to NIE-NPs and CNIE-NPs for 6 h followed by incubation with fresh medium without nanoparticles for 18 h to show long retention of NIE and short retention of CNIE. Scale bar is 10 μm. (c) Representative SEM images of untreated 4T1 tumor and 4T1 tumor cells treated with NIE-NPs for 6 h. Scale bar is 10 μm. The concentration of NIE-NPs was 50 μM. (d and e) (d) Cellular fluorescence distribution images and (e) representative SEM images of murine CD8 T cells (isolated from mouse spleen, CellTracker green CMFDA dye labeled, green fluorescence) after incubation with esterase-pretreated NIE-NPs to show NIE around cells. α_4_β_1_ integrins on the surface of murine CD8 T cells were preactivated by Mn^2+^ (1 mM). Scale bar is 10 μm. (f) Experimental scheme and cellular fluorescence distribution images of NIE-NPs (fluorescent red), after interaction with 4T1 tumor and murine CD8 T cells (fluorescent green, α_4_β_1_ integrins were preactivated by Mn^2+^). It shows that nanofibrillar networks (bispecific NIE) cover 4T1 tumor cells, which in turn capture CD8 T cells. More incubation time, more bound CD8 T cells. Scale bar is 10 μm. (g) Representative SEM images of 4T1 tumor and CD8 T cells after treatment with NIE-NPs (see part f). (h) Representative images of M2-like murine macrophages and subsequent re-education by NIE-NFs, NIE-NFs plus esterase, or R848 at different time points. Scale bar is 20 μm. Statistical significance was calculated using one-way ANOVA followed by Tukey’s post hoc analysis: *P < 0.05, **P < 0.01, ***P < 0.001.

To simulate the processes of initial fibrillar transformation of NIE-NPs on the surface of 4T1 tumor cells followed by T cell binding, we incubated 4T1 tumor cells with NIE-NPs for 6 h; unbound NIE-NPs were then washed off, followed by addition of fresh medium containing esterase but without NIE-NPs. After 1 h of incubation, murine CD8 T cells with α_4_β_1_ integrins activated by Mn^2+^ were added and incubated with 4T1 tumor cells for 2 or 4 h. After that, unbound CD8 T cells were gently removed prior to CLSM imaging (Figure 2f). As expected, a fibrillar structure layer with red fluorescence was detected surrounding the surface of the 4T1 tumor cells, and the CD8 T cells (green fluorescence) were found to interact with the red fluorescent fibrillar network and in close proximity to the 4T1 tumor cells, after 2 h of incubation. As the incubation time was increased to 4 h, many more T cells were found around the 4T1 tumor cells, which was consistent with our notion that the nanofibrillar-based bispecific NIE would facilitate the capturing and retention of immune cells. SEM imaging provided critical evidence that the bispecific NIE had played a significant role in the direct physical contact between 4T1 tumor cells and T cells through the nanofibrillar network (Figure 2g). The conversion of tumor-associated macrophages (TAMs) from an immunosuppressive M2-polarized phenotype to an anti-tumor M1-polarized phenotype is one of the major immunotherapeutic strategies for reprogramming the immunosuppressive TME.^26^ Addition of esterase to the culture medium followed by 24 h of incubation resulted in morphological change of the M2-state toward the round and flattened M1-state, a decrease in Arg1 and Mrc1, and an increase in IL-12 and Nos2 expression (Figures 2h and S11).

NIE-NPs were found to be nontoxic: blood counts, platelets, creatinine, and liver function tests obtained from normal Balb/c mice treated with eight consecutive q.o.d. intravenous (i.v.) doses of NIE-NPs were within normal limits (Figures S12 and S13). *In vivo* blood pharmacokinetics (PK) studies in rats showed that NIE-NPs possessed a long circulation time (T-half (α): 2.866 h and T-half (β): 23.186 h), indicating their stability during circulation (Figure S14). For biodistribution studies, NIE-NPs were tail vein injected once into Balb/c mice bearing syngeneic orthotopic 4T1 breast cancer; 10, 24, 48, 72, 120, and 168 h later, tumor and main organs were excised for *ex vivo* fluorescent imaging (Figure 3a and b). A significant fluorescent signal of *Pa* was found to persist in tumor tissue for over 168 h, while the fluorescent signal in normal organs began to decline after 10 h and was almost undetectable in the main organs at 72 h. In sharp contrast, the fluorescent signal of *Pa* at tumor tissue treated by CNIE-NPs was found to gradually decline over time after peaking at 24 h (Figures 3c and S15). The prolonged retention of the fluorescent signal in NIE-NPs-treated mice could be attributed to *in situ* receptor-mediated transformation of NIE-NPs into NIE-NFs at the TME (Figure 3d). TEM studies on excised tumor sections, 72 h after i.v. administration, showed abundant bundles of nanofibrils in the extracellular matrix while no such nanofibrils were observed in negative CNIE-NPs-treated and untreated mice (Figure 3e). H&E staining and TEM imaging of normal organs showed normal histology without any pathologic changes, and there was no sign of fibrillar structures (Figure S16). Fluorescent micrographs of the tumors and overlying skin revealed an intense fluorescent signal in the tumor region but negligible signal in the normal skin (Figure 3f). The tumor tissue distribution of R848 over time was also determined with high-pressure liquid chromatography–mass spectroscopy (HPLC-MS, Figure 3g). We found that the R848 uptake by the tumor was quite high at 24 h (3.62 μg per g tissue) and that about one-third of the R848 was found to be retained at the tumor site (1.14 μg per g tissue) even at 7 days.

**Figure 3:**
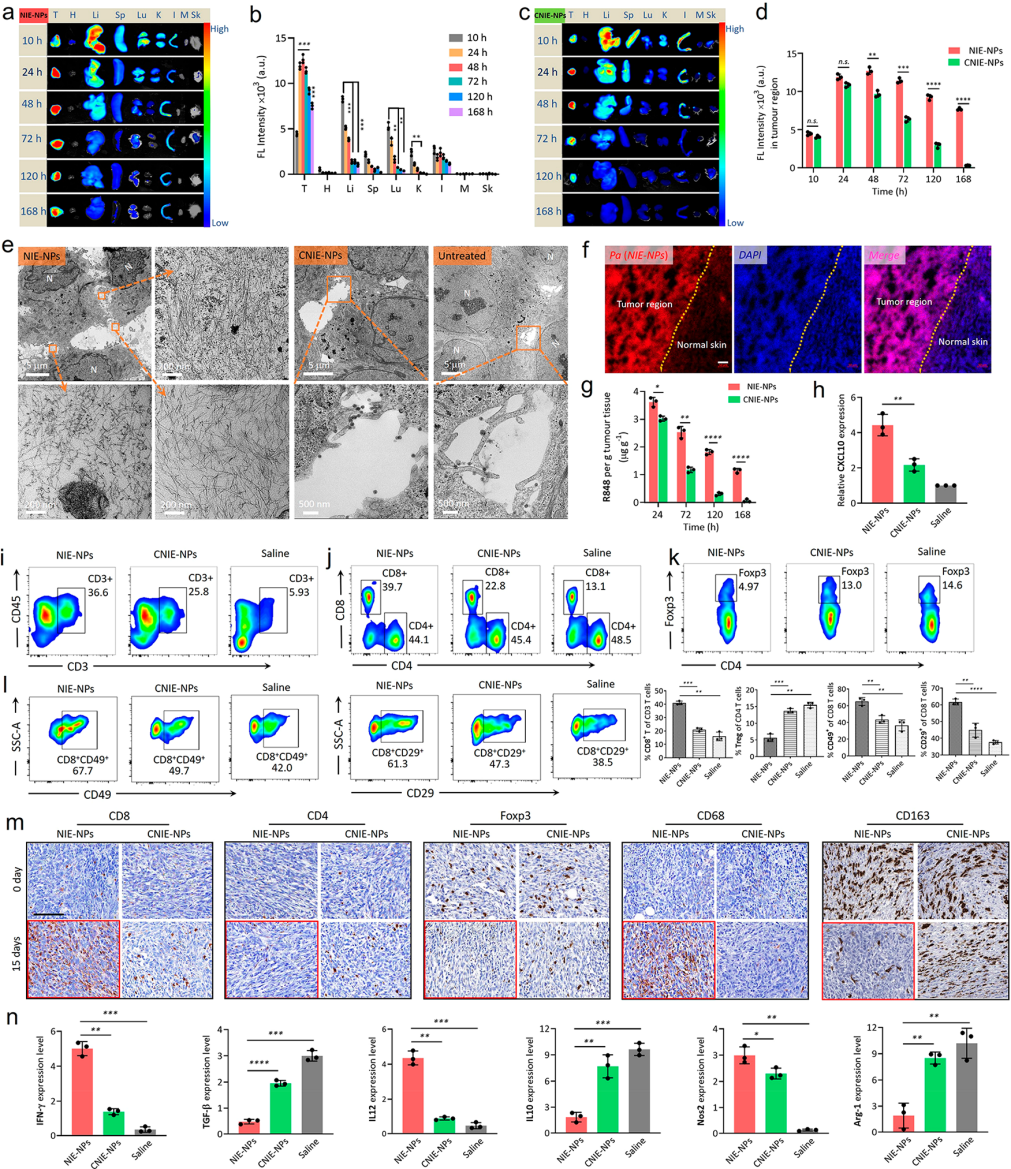
*In vivo* evaluation of NIE targeting tumor cells, and *in situ* nanofibrillar transformation to capture T_eff_ cell and facilitate the long-term retention and activate immunity. (a and b) Time-dependent *ex vivo* fluorescence (FL) images (a) and quantitative analysis (b) of tumor tissues and major organs (heart (H), liver (Li), spleen (Sp), lung (Lu), kidney (K), intestine (I), muscle (M), and skin (Sk)) collected at 10, 24, 48, 72, 120, and 168 h post-injection of NIE-NPs. Data are presented as mean ± s.d., *n* = 3 independent experiments. (c) Time-dependent *ex vivo* fluorescence images of tumor tissues collected at 10, 24, 48, 72, 120, and 168 h post-injection of CNIE-NPs. (d) Fluorescence (FL) quantification of tumor tissues collected at 10, 24, 48, 72, 120, and 168 h post-injection of NIE-NPs and CNIE-NPs. Data are presented as mean ± s.d., *n* = 3 independent experiments. (e) Representative TEM images of distribution in tumor tissue and *in situ* fibrillar transformation of NIE-NPs, CNIE-NPs, and the untreated control group at 72 h post-injection. “N” depicts nucleus. (f) Fluorescence distribution images of NIE-NPs in the tumor tissue region and normal skin tissue at 72 h post-injection (red, *Pa* of NIE-NPs; blue, DAPI; scale bars, 50 μm). (g) R484 distribution retention in tumor tissues at different time points post-injection of NIE-NPs and CNIE-NPs. Injection dose of R848:0.94 mg kg^–1^; data were mean ± s.d., *n* = 3 for each time point. (h) Expression of CXCL10 chemokine within the tumor tissues after 5 days of NIE-NPs, CNIE-NPs, and saline treatment (*n* = 3; data were mean ± s.d.; single injection). (i) Representative flow cytometric analysis images and corresponding quantification of CD45^+^CD3^+^, CD8^+^/CD4^+^, CD4^+^Foxp3^+^, CD8^+^CD49^+^, and CD8^+^CD29^+^ T cells within the 4T1 tumors excised from mice treated with NIE-NPs, CNIE-NPs, or the saline control. (j) Immunohistochemistry (IHC) of tumors excised from mice after treatment with NIE-NPs or CNIE-NPs. Representative images are shown for the IHC staining of T cells (CD8^+^, CD4^+^, and Foxp3^+^) and macrophage markers (CD68 and CD163). Scale bar is 100 μm. (k) The expression levels (qPCR assay) of IFN-γ, TGF-β, IL12, IL10, Nos2, and Arg-1 in 4T1 tumors excised from mice 15 days after treatment with NIE-NPs or CNIE-NPs (*n* = 3; data were mean ± s.d.). Statistical significance was calculated using a two-sided unpaired *t* test compared to the NIE-NPs group: *P < 0.05, **P < 0.01, ***P < 0.001, ****P < 0.0001.

We evaluated whether the bispecific NIE (single injection) could capture T_eff_ cells and facilitate their long-term retention at the tumor sites and re-educate TAMs *in vivo*. First, NIE-NPs were found to significantly stimulate the production of chemokine CXCL10 at the tumor site (Figure 3h), which was known to facilitate recruitment of T_eff_ cells.^32,33^ This should be attributed to sustained release of R848, which is known to induce the production of chemokines.^34−36^ We confirmed that the proportion of CD45^+^CD3^+^ and CD45^+^CD3^+^CD8^+^ T cells in the NIE-NPs-treated tumor tissue was substantially higher than those from mice treated with endocytic CNIE-NPs or saline (Figures 3i and S17). Second, the relative abundance of CD4^+^Foxp3^+^ T_regs_ at the tumor site was substantially lower in mice that received the NIE-NPs treatment than that in mice treated with CNIE-NPs, i.e., 4.97% vs 13.0%. We also confirmed the higher expression (CD49^+^) and activation (CD29^+^) of α_4_β_1_ integrin in tumor-infiltrating CD8 T cells after NIE-NPs treatment, which were conducive to capturing of CD8 T cells by LLP2A displayed at the TME. Third, IHC staining of tumor sections demonstrated an increase in M1-polarized macrophage marker CD68 and a decrease in M2-polarized macrophage marker CD163 in the NIE-NPs-treated group (Figure 3j). Fourth, the high expression levels of IFN-γ, IL-12, and Nos2 and low expression levels of TGF-β, IL-10, and Arg-1 in the tumor tissue confirmed that a strong tumor-specific immune response had been elicited (Figure 3k).

We evaluated the roles of T cell capturing ligand (LLP2A) and TLR7/8 immunoagonist (R848), the two components of NIE-NPs, in inducing chemokine production and T cell capturing at the TME *in vivo*. As shown in Figure 4a and b, R848 (group 2) was more effective in inducing chemokine secretion than the LLP2A ligand (group 3), but the population of CD8 T cells in group 3 was slightly higher than that in group 2. This suggested that LLP2A ligands should have played a significant role in capturing and retention of CD8 T cells at the TME, more so than R848. However, LLP2A or R848, each alone, was found to be insufficient to induce a robust antitumor immune response. It is clear that group 4 mice treated with both LLP2A and R848 were able to mount a robust antitumor immune response, resulting in a higher level of chemokine expression, more CD8 T cells, and fewer T_reg_ cells.

**Figure 4:**
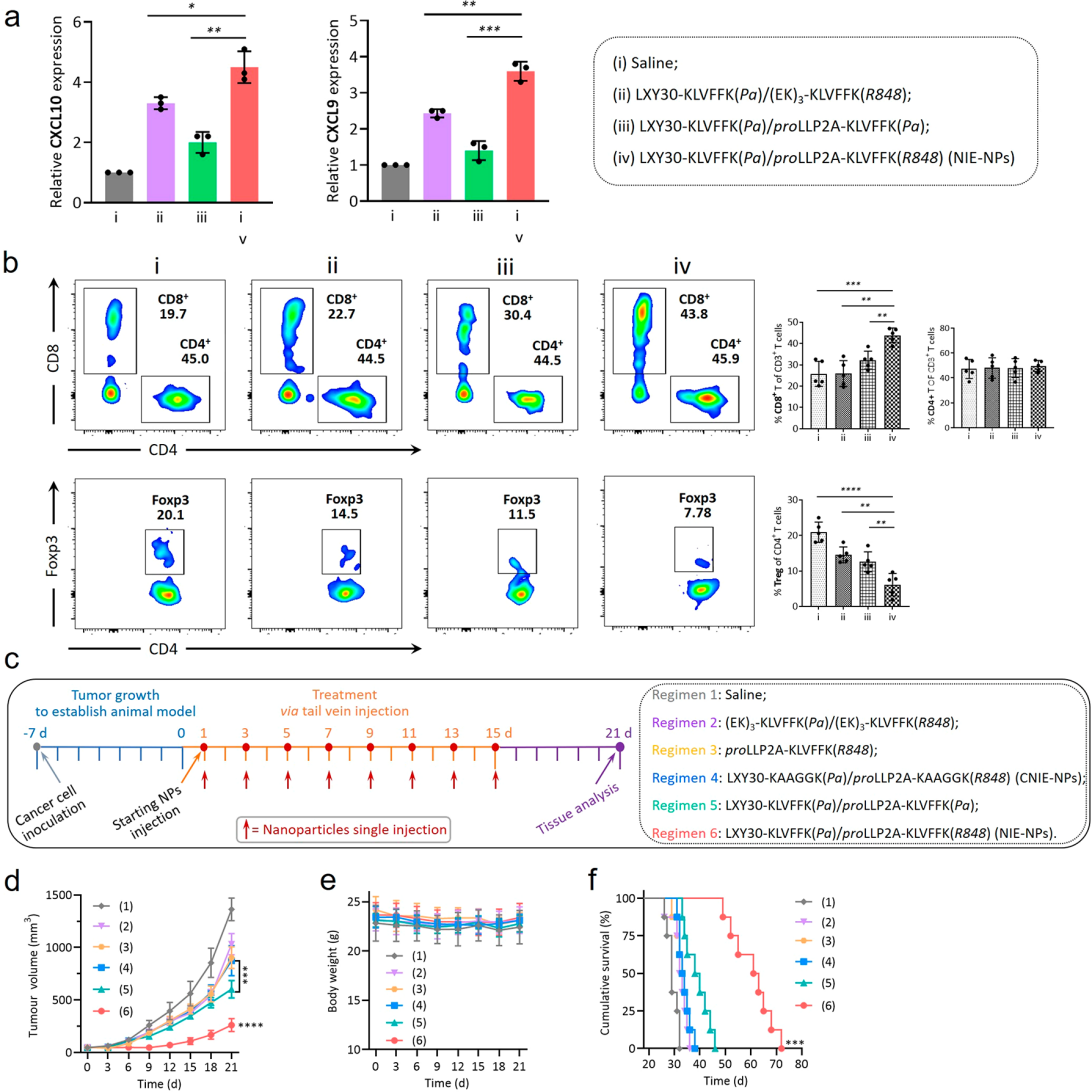
Intrinsic antitumor immune efficacy of bispecific NIE in Balb/c mice bearing 4T1 breast tumor. (a) Expression of CXCL10 and CXCL9 chemokine on day 7 within the excised tumor tissues of mice after different treatments (13 mg/kg each dose, every other day; *n* = 3; data were mean ± s.d.). (b) Representative flow cytometric analysis images and relative quantification of CD8^+^/CD4^+^ and CD4^+^Foxp3^+^ T cells within the 4T1 tumors excised from mice after different treatments at day 15. All treatment regimens were tail vein injected consecutively three times q.o.d. (13 mg/kg each dose, every other day, total three times; *n* = 5; data were mean ± s.d.). In parts a and b, (1) saline; (2) LXY30-KLVFFK(*Pa*)/(EK)3-KLVFFK(*R848*) (fibrillar-transformation but absence of LLP2A ligand); (3) LXY30-KLVFFK(*Pa*)/proLLP2A-KLVFFK(*Pa*) (fibrillar-transformation but absence of R848); and (4) LXY30-KLVFFK(*Pa*)/proLLP2A-KLVFFK(*R848*) (complete NIE-NPs). (c) Experimental design: orthotopic tumor inoculation and treatment protocol; regimen 6 is NIE-NPs with all four critical components: (1) saline; (2) (EK)3-KLVFFK(*Pa*)/(EK)3-KLVFFK(*R848*) (“R848 only” in micellar formulation); (3) *pro*LLP2A-KLVFFK(*R848*) (single monomer); (4) LXY30-KAAGGK(*Pa*)/proLLP2A-KAAGGK(*R848*) (untransformable negative control CNIE-NPs); (5) LXY30-KLVFFK(*Pa*)/proLLP2A-KLVFFK(*Pa*) (fibrillar-transformation but absence of R848); (6) LXY30-KLVFFK(*Pa*)/proLLP2A-KLVFFK(*R848*). (d and e) Observation of the tumor inhibitory effect (d) and weight change (e) of mice bearing orthotopic 4T1 tumor over 21 d after initiation of treatment (*n* = 8 per group). Data are presented as mean ± s.d. (f) Cumulative survival of different treatment groups of mice bearing 4T1 breast tumors. Data are presented as mean ± s.d. Statistical significance was calculated using one-way ANOVA followed by Tukey’s post hoc analysis: *P < 0.05, **P < 0.01, ***P < 0.001, ****P < 0.0001.

A therapeutic efficacy study of a programmable bispecific NIE was performed in syngeneic orthotopic 4T1 breast cancer-bearing mice (Figure 4c–f). Regimen 6 is the complete NIE-NPs, containing all four critical components: LXY30, *pro*LLP2A, R848, and KLVFF, whereas regimen 2, 3, 4, or 5 each lacks some components of NIE-NPs. Regimen 6 (NIE-NPs) was found to be the most efficacious with significant tumor growth suppression and prolonged survival, indicating the importance of the combination of the homing strategy of T cells and sustained release of TLR7/8 agonist. Regimen 5 (fibrillar-transformation but no R848) demonstrated significant tumor suppression compared to groups 2, 3, and 4. None of the mice in this therapeutic study showed any symptoms of dehydration or significant body weight loss during the entire treatment period. Regimen 6 showed a great increase in the frequency of CD3^+^ and CD8^+^ T cells within the tumors and was the most efficacious in restoring the immunoactive state of the TME (Figure S18).^10,35^ Tumor sections (H&E) obtained from mice treated with NIE-NPs revealed a marked decrease in *K*_i_-67 expression, an increase in CD8^+^ T cells, and a decrease in Foxp3 (T_reg_ cells), compared with other control groups. There was an increase in CD68 and a decrease in CD163, and the percentage of M1-phenotype macrophage in the total macrophages was 60%, which was much higher than that of the other control groups (Figures S19 and S20). To better clarify the contribution of the recruitment of CD8 T cells to tumors in NIE-NPs’ immunotherapy, we performed a tumor protection experiment with depleting the CD8 T cell antibody (Figure S21). As expected, the therapeutic efficacy of NIE-NPs was diminished in mice given CD8 T cell-depleting antibody (poor tumor inhibition and short survival), indicating the effectiveness and importance of tumor-homing of CD8 T cells in NIE therapy.

Since the receptor-mediated bispecific NIE could significantly mount a systemic antitumor response by capturing T_eff_ cells and reprogramming of the TME, we believe it could enhance the efficacy of ICB therapy. We investigated the therapeutic synergy between the bispecific NIE and PD-1/PD-L1 ICB therapy (Figure 5a–c). Not unexpectedly, regimen 5 plus anti-PD-1 treatment significantly suppressed tumor growth, resulting in a longer median survival, compared with eight treatments of regimen 5 without anti-PD-1 as shown in Figure 4d and f (49.5 d vs 39 d); both of these treatments, however, were not able to completely eliminate the tumors. Most remarkably, mice treated with regimen 6 (NIE-NPs) plus anti-PD-1 resulted in gradual shrinkage and eventual complete elimination of tumors within 21 days, and without any sign of recurrence during the observation period of 90 days, validating the synergistic effects of our bispecific NIE with ICB therapy.

**Figure 5:**
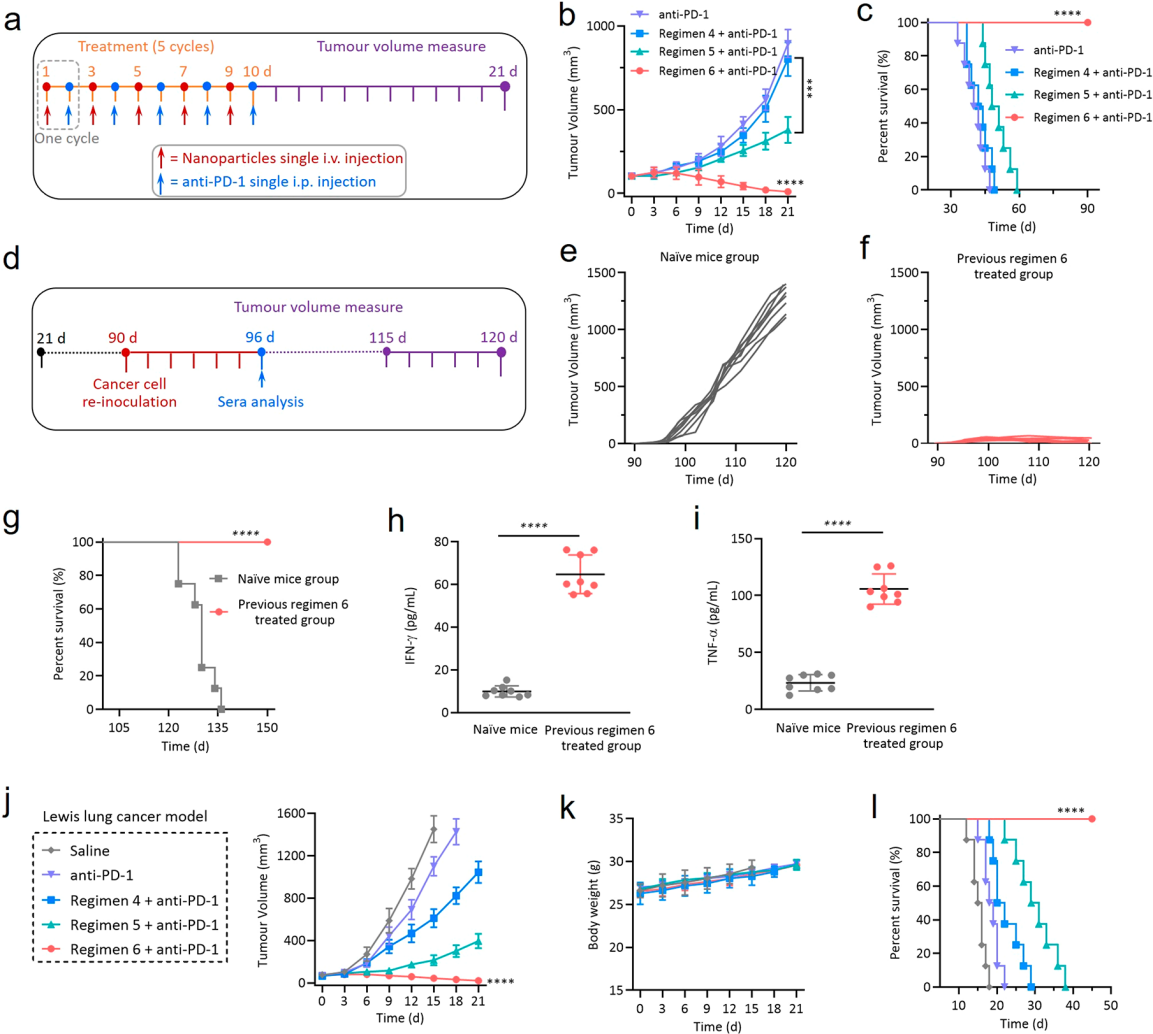
Bispecific NIE greatly enhances ICB therapy in mice bearing 4T1 breast tumor and Lewis lung tumor. (a) Experimental design: orthotopic tumor inoculation and treatment protocol (four treatment arms; regimens 4, 5, and 6 are the same as those shown in Figure 4a). (b) Tumor response in mice bearing orthotopic 4T1 tumor over 21 d of treatment (*n* = 8 per group). Data are presented as mean ± s.d. (c) Cumulative survival of the four treatment groups. (d) Experimental design: Mice previously treated with regimen 6 plus anti-PD-1 Ab were rechallenged with reinoculation of 4T1 breast cancer cells on day 90. The same operation was carried out on the same age naïve mice as a negative control group. (e) No antitumor immune memory effect was observed in the same age naïve mice. (f) An antitumor immune memory effect was observed in mice previously treated with regimen 6 and anti-PD-1 Ab. (g) Cumulative survival of naïve mice and previously regimen 6 plus anti-PD-1-treated mice. (h and i) IFN-γ (h) and TNF-α (i) level in mice sera 6 days after mice were rechallenged with 4T1 tumor cells. (j and k) Observation of tumor inhibitory effect (j) and weight change (k) of mice bearing subcutaneous murine Lewis lung tumor over 21 d after initiation of treatment (*n* = 8 per group). The treatment protocol followed the experimental design in part a; five cycles (i.v. regimens 4–6 and i.p. anti-PD-1). Data are presented as mean ± s.d. (l) Cumulative survival of different treatment groups of mice bearing subcutaneous murine Lewis lung tumors. Statistical significance was calculated using one-way ANOVA followed by Tukey’s post hoc analysis: *P < 0.05, **P < 0.01, ***P < 0.001, ****P < 0.0001.

To assess whether the synergistic therapy of bispecific NIE plus anti-PD-1 Ab could induce a memory response, we rechallenged the “cured” mice from the previous experiment (regimen 6 plus anti-PD-1treatment, Figure 5a–c) with 4T1 tumor cells on the opposite mammary fat pad on day 90; mice of the same age were used as a negative control (Figure 5d–g). Either no tumor growth or a significant delay in tumor growth was observed in mice previously treated successfully with NIE-NPs plus anti-PD-1. The survival curves of this experimental group correlated well with the tumor growth results. In addition, the serum levels of cytokines such as TNF-α and IFN-γ in this experimental group were found to be much higher after the rechallenge with 4T1 tumor cells for 6 days (Figure 5h and i). These results suggest that a durable and robust T cell memory response was generated by regimen 6 (NIE-NPs) plus anti-PD-1 given previously. In addition, we speculated that, like the 4T1 breast cancer model, the NIE would also synergize ICB treatment of the lung cancer model. As expected, complete tumor regression and prolonged survival were obtained for the treatment of the Lewis lung syngeneic subcutaneous murine tumor model using NIE-NPs plus anti-PD-1 (five cycles, Figure 5j–l). No systemic toxicity and weight loss were detected.

In spite of the clinical success of ICB therapy, only a fraction of cancer patients benefit from it. Defects in infiltration of T_eff_ cells to the tumor sites are probably one of the main reasons why many patients remain refractory to such treatment.^3^ We believe the receptor-mediated bispecific NIE reported here can provide a relatively simple solution to this challenge. By incorporating pro-ligands LLP2A and R848 to the *in vivo* transformable nanofibrillar networks, we have already demonstrated in syngeneic 4T1 breast cancer and Lewis lung cancer models that this nontoxic treatment can (1) significantly enhance the capturing of T_eff_ cells (CD8^+^ T cells) in the tumor region, (2) promote retention of T cells at close proximity to the tumor cells, and (3) provide sustained release of R848 at the TME, resulting in the significant enhancement of the efficacy of the anti-PD1 antibody-based ICB. Since the programmable bispecific NIE is modular, we have the options of combinatorially incorporating various ligands, pro-ligands, or immunomodulators to generate a series of nanoengagers that may be applied for capturing other beneficial antitumor immune cells.

## Supporting Information Available

Additional details on experimental materials and methods. Figures showing chemical characterization of peptide monomers, NIE-NPs and NIE-NFs, interaction between NPs and living cells, NPs’ treatment, and immunity index. (PDF)

## References

[ref1] Postow M. A.; Sidlow R.; Hellmann M. D. Immune-related adverse events associated with immune checkpoint blockade. New. Engl. J. Med. 2018, 378, 158–168. 10.1056/NEJMra1703481. 29320654

[ref2] Topalian S. L.; Taube J. M.; Anders R. A.; Pardoll D. M. Mechanism-driven biomarkers to guide immune checkpoint blockade in cancer therapy. Nat. Rev. Cancer 2016, 16, 275–287. 10.1038/nrc.2016.36. 27079802 PMC5381938

[ref3] Sackstein R.; Schatton T.; Barthel S. R. T-lymphocyte homing: an underappreciated yet critical hurdle for successful cancer immunotherapy. Lab. Invest. 2017, 97, 669–697. 10.1038/labinvest.2017.25. 28346400 PMC5446300

[ref4] Fares C. M.; Allen E. M. V.; Drake C. G.; Allison J. P.; Hu-Lieskovan S. Mechanisms of resistance to immune checkpoint blockade: why does checkpoint inhibitor immunotherapy not work for all patients?. ASCO Educational Book 2019, 39, 147–164. 10.1200/EDBK_240837. 31099674

[ref5] Riley R. S.; June C. H.; Langer R.; Mitchell M. J. Delivery technologies for cancer immunotherapy. Nat. Rev. Drug Discovery 2019, 18, 175–196. 10.1038/s41573-018-0006-z. 30622344 PMC6410566

[ref6] Zhang C.; Pu K. Molecular and nanoengineering approaches towards activatable cancer immunotherapy. Chem. Soc. Rev. 2020, 49, 4234–4253. 10.1039/C9CS00773C. 32452475

[ref7] Shi J.; Kantoff P. W.; Wooster R.; Farokhzad O. C. Cancer nanomedicine: progress, challenges and opportunities. Nat. Rev. Cancer 2017, 17, 20–37. 10.1038/nrc.2016.108. 27834398 PMC5575742

[ref8] Nam J.; Son S.; Park K. S.; Zou W.; Shea L. D.; Moon J. J. Cancer nanomedicine for combination cancer immunotherapy. Nat. Rev. Mater. 2019, 4, 398–414. 10.1038/s41578-019-0108-1.

[ref9] Jain R. K.; Stylianopoulos T. Delivering nanomedicine to solid tumors. Nat. Rev. Clin. Oncol. 2010, 7, 653–664. 10.1038/nrclinonc.2010.139. 20838415 PMC3065247

[ref10] Xiao Z.; Su Z.; Han S.; Huang J.; Lin L.; Shuai X. Dual pH-sensitive nanodrug blocks PD-1 immune checkpoint and uses T cells to deliver NF-κB inhibitor for antitumor immunotherapy. Sci. Adv. 2020, 6, eaay7785 10.1126/sciadv.aay7785. 32076650 PMC7002126

[ref11] Zhang L.; Jing D.; Jiang N.; Rojalin T.; Baehr C. M.; Zhang D.; Xiao W.; Wu Y.; Cong Z.; Li J. J.; Li Y.; Wang L.; Lam K. S. Transformable peptide nanoparticles arrest HER2 signalling and cause cancer cell death in vivo. Nat. Nanotechnol. 2020, 15, 145–153. 10.1038/s41565-019-0626-4. 31988501 PMC7147967

[ref12] Li Y.; Wang Y.; Huang G.; Gao J. Cooperativity principles in self-assembled nanomedicine. Chem. Rev. 2018, 118, 5359–5391. 10.1021/acs.chemrev.8b00195. 29693377 PMC6524957

[ref13] Wang H.; Feng Z.; Xu B. Assemblies of peptides in a complex environment and their applications. Angew. Chem., Int. Ed. 2019, 58, 10423–10432. 10.1002/anie.201814552. PMC6656613 30903643

[ref14] Cheng K.; Ding Y.; Zhao Y.; Ye S.; Zhao X.; Zhang Y.; Ji T.; Wu H.; Wang B.; Anderson G. J. Sequentially responsive therapeutic peptide assembling nanoparticles for dual-targeted cancer immunotherapy. Nano Lett. 2018, 18, 3250–3258. 10.1021/acs.nanolett.8b01071. 29683683

[ref15] Yang P.-P.; Zhang K.; He P.-P.; Fan Y.; Gao X. J.; Gao X.; Chen Z.; Hou D.; Li Y.; Yi Y.; Cheng D.; Zhang J.; Shi L.; Zhang X.; Wang L.; Wang H. A biomimetic platelet based on assembling peptides initiates artificial coagulation. Sci. Adv. 2020, 6, eaaz4107 10.1126/sciadv.aaz4107. 32766439 PMC7385434

[ref16] Luo Q.; Lin Y.; Yang P.; Wang Y.; Qi G.; Qiao Z.; Li B.; Zhang K.; Zhang J.; Wang L.; Wang H. A self-destructive nanosweeper that captures and clears amyloid β-peptides. Nat. Commun. 2018, 9, 1802 10.1038/s41467-018-04255-z. 29728565 PMC5935695

[ref17] Xiao W.; Li T.; Bononi F. C.; Lac D.; Kekessie I. A.; Liu Y.; Sanchez E.; Mazloom A.; Ma A.; Lin J.; Tran J.; Yang K.; Lam K. S.; Liu R. Discovery and characterization of a high-affinity and high-specificity peptide ligand LXY30 for in vivo targeting of α3 integrin-expressing human tumors. EJNMMI Res. 2016, 6, 18 10.1186/s13550-016-0165-z. 26922417 PMC4769701

[ref18] Liu M.; Zhang Y.; Yang J.; Cui X.; Zhou Z.; Zhan H.; Ding K.; Tian X.; Yang Z.; Fung K.-M. A.; Edil B. H.; Postier R. G.; Bronze M. S.; Fernandez-Zapico M. E.; Stemmler M. P.; Brabletz T.; Li Y.-P.; Houchen C. W.; Li M. ZIP4 Increases Expression of Transcription Factor ZEB1 to Promote Integrin α_3_β_1_ Signaling and Inhibit Expression of the Gemcitabine Transporter ENT1 in Pancreatic Cancer Cells. Gastroenterology 2020, 158, 679–692. 10.1053/j.gastro.2019.10.038. 31711924 PMC7837454

[ref19] Xiao W.; Ma W.; Wei S.; Li Q.; Liu R.; Carney R. P.; Yang K.; Lee J.; Nyugen A.; Yoneda K. Y.; Lam K. S.; Li T. High-affinity peptide ligand LXY30 for targeting α3β1 integrin in non-small cell lung cancer. J. Hematol Oncol. 2019, 12, 56 10.1186/s13045-019-0740-7. 31182116 PMC6558829

[ref20] Yang P. P.; Luo Q.; Qi G.; Gao Y.; Li B.; Zhang J.; Wang L.; Wang H. Host materials transformable in tumor microenvironment for homing theranostics. Adv. Mater. 2017, 29, 1605869 10.1002/adma.201605869. 28195446

[ref21] Tan J.; Town T.; Crawford F.; Mori T.; DelleDonne A.; Crescentini R.; Obregon D.; Flavell R. A.; Mullan M. J. Role of CD40 ligand in amyloidosis in transgenic Alzheimer’s mice. Nat. Neurosci. 2002, 5, 1288–1293. 10.1038/nn968. 12402041

[ref22] Hock C.; Konietzko U.; Papassotiropoulos A.; Wollmer A.; Streffer J.; von Rotz R. C.; Davey G.; Moritz E.; Nitsch R. M. Generation of antibodies specific for β-amyloid by vaccination of patients with Alzheimer disease. Nat. Med. 2002, 8, 1270–1275. 10.1038/nm783. 12379846

[ref23] Peng L.; Liu R.; Marik J.; Wang X.; Takada Y.; Lam K. S. Combinatorial chemistry identifies high-affinity peptidomimetics against α_4_β_1_ integrin for in vivo tumor imaging. Nat. Chem. Biol. 2006, 2, 381–389. 10.1038/nchembio798. 16767086

[ref24] Wang C.; Wang J.; Zhang X.; Yu S.; Wen D.; Hu Q.; Ye Y.; Bomba H.; Hu X.; Liu Z.; Dotti G.; Gu Z. *In situ* formed reactive oxygen species-responsive scaffold with gemcitabine and checkpoint inhibitor for combination therapy. Sci. Transl. Med. 2018, 10, eaan3682 10.1126/scitranslmed.aan3682. 29467299

[ref25] Pardoll D. M. The blockade of immune checkpoints in cancer immunotherapy. Nat. Rev. Cancer 2012, 12, 252–264. 10.1038/nrc3239. 22437870 PMC4856023

[ref26] Sharma P.; Allison J. P. The future of immune checkpoint therapy. Science 2015, 348, 56–61. 10.1126/science.aaa8172. 25838373

[ref27] Zhang L.; Jing D.; Wang L.; Sun Y.; Li J. J.; Hill B.; Yang F.; Li Y.; Lam K. S. Unique photochemo-immuno-nanoplatform against orthotopic xenograft oral cancer and metastatic syngeneic breast cancer. Nano Lett. 2018, 18, 7092–7103. 10.1021/acs.nanolett.8b03096. 30339018 PMC6501589

[ref28] Keller N.; Calik M.; Sharapa D.; Soni H. R.; Zehetmaier P. M.; Rager S.; Auras F.; Jakowetz A. C.; Goerling A.; Clark T.; Bein T. Enforcing extended porphyrin J-aggregate stacking in covalent organic frameworks. J. Am. Chem. Soc. 2018, 140, 16544–16552. 10.1021/jacs.8b08088. 30392360 PMC6400425

[ref29] Cheng M. H.; Harmatys K. M.; Charron D. M.; Chen J.; Zheng G. Stable J-aggregation of an aza-BODIPY-lipid in a liposome for optical cancer imaging. Angew. Chem., Int. Ed. 2019, 131, 13528–13533. 10.1002/ange.201907754. 31344292

[ref30] Maiti N. C.; Mazumdar S.; Periasamy N. J-and H-aggregates of porphyrin-surfactant complexes: time-resolved fluorescence and other spectroscopic studies. J. Phys. Chem. B 1998, 102, 1528–1538. 10.1021/jp9723372.

[ref31] Korevaar P. A.; George S. J.; Markvoort A. J.; Smulders M. M. J.; Hilbers P. A. J.; Schenning A. P. H. J.; De Greef T. F. A.; Meijer E. W. Pathway complexity in supramolecular polymerization. Nature 2012, 481, 492–496. 10.1038/nature10720. 22258506

[ref32] Wang F.; Xu D.; Su H.; Zhang W.; Sun X.; Monroe M. K.; Chakroun R. W.; Wang Z.; Dai W.; Oh R.; Wang H.; Fan Q.; Wan F.; Cui H. Supramolecular prodrug hydrogelator as an immune booster for checkpoint blocker-based immunotherapy. Sci. Adv. 2020, 6, eaaz8985 10.1126/sciadv.aaz8985. 32490201 PMC7239700

[ref33] Park C. G.; Hartl C. A.; Schmid D.; Carmona E. M.; Kim H. J.; Goldberg M. S. Extended release of perioperative immunotherapy prevents tumor recurrence and eliminates metastases. Sci. Transl. Med. 2018, 10, eaar1916 10.1126/scitranslmed.aar1916. 29563317

[ref34] Lynn G. M.; Laga R.; Darrah P. A.; Ishizuka A. S.; Balaci A. J.; Dulcey A. E.; Pechar M.; Pola R.; Gerner M. Y.; Yamamoto A.; Buechler C. R.; Quinn K. M.; Smelkinson M. G.; Vanek O.; Cawood R.; Hills T.; Vasalatiy O.; Kastenmüller K.; Francica J. R.; Stutts L.; Tom J. K.; Ryu K. A.; Esser-Kahn A. P.; Etrych T.; Fisher K. D.; Seymour L. W.; Seder R. A. In vivo characterization of the physicochemical properties of polymer-linked TLR agonists that enhance vaccine immunogenicity. Nat. Biotechnol. 2015, 33, 1201–1210. 10.1038/nbt.3371. 26501954 PMC5842712

[ref35] Asselin-Paturel C.; Brizard G.; Chemin K.; Boonstra A.; O’Garra A.; Vicari A.; Trinchieri G. Type I interferon dependence of plasmacytoid dendritic cell activation and migration. J. Exp. Med. 2005, 201, 1157–1167. 10.1084/jem.20041930. 15795237 PMC2213121

[ref36] Grinberg-Bleyer Y.; Oh H.; Desrichard A.; Bhatt D. M.; Caron R.; Chan T. A.; Schmid R. M.; Klein U.; Hayden M. S.; Ghosh S. NF-κB c-Rel is crucial for the regulatory T cell immune checkpoint in cancer. Cell 2017, 170, 1096–1108002Ey2. 10.1016/j.cell.2017.08.004. 28886380 PMC5633372

