# Supplementary material for: Programmable Bispecific Nano-immunoengager That Captures T Cells and Reprograms Tumor Microenvironment
Source: Nano Lett. 2022 Aug 4;22(17):6866–76. doi: 10.1021/acs.nanolett.2c00582 (PMC9479133; doi:10.1021/acs.nanolett.2c00582)
Supplement: Supplementary file 1 — nl2c00582_si_001.pdf [file nl2c00582_si_001.pdf]

Supplementary Information

A programmable bispecific nano-immuno-engager promotes T cell homing and reprograms tumor microenvironment

Lu Zhang[1,2,*], Ruonan Bo[1], Yi Wu[1], Longmeng Li[1], Zheng Zhu[1], Ai-Hong Ma[1], Wenwu Xiao[1], Yanyu Huang[1], Tatu Rojalin[1], Xingbin Yin[1], Chunping Mao[2], Fengyi Wang[2], Yongheng Wang[1], Hongyong Zhang[1], Kelmen E. Low[1], Kiana Lee[1], Yousif Ajena[1], Di Jing[1], Dalin Zhang[1], Christopher M. Baehr[1], Ruiwu Liu[1], Lei Wang[3,*], Yuanpei Li[1,*] and Kit S. Lam[1,4,*]

[1]Department of Biochemistry and Molecular Medicine, UC Davis NCI-designated Comprehensive Cancer Center, University of California Davis, Sacramento, CA, 95817, USA.

[2]Department of Biomedical Engineering, Southern University of Science and Technology, Shenzhen, Guangdong, 518055, China.

[3]CAS Center for Excellence in Nanoscience, CAS Key Laboratory for Biomedical Effects of Nanomaterials and Nanosafety, National Center for Nanoscience and Technology, Beijing, 100190, China.

[4]Division of Hematology and Oncology, Department of Internal Medicine, School of Medicine, University of California Davis, Sacramento, CA, 95817, USA.

*e-mail: kslam@ucdavis.edu; zhanglu@sustech.edu.cn; lypli@ucdavis.edu; wanglei@nanoctr.cn.

Materials and Methods

Preparation of transformable peptide monomers (TPMs). All TPMs were synthesized by standard solid-phase peptide synthesis techniques. LXY30-KLVFFK(Dde)-Beads, LXY30-KAAGGK(Dde)-Beads, LLP2A-KLVFFK(Dde)-Beads and LLP2A-KAAGGK(Dde)-Beads were firstly synthesized in sequence. Then amino protection group (Dde) was removed for 5 h using the mixed solution of imidazole and hydroxylamine (the ratio was 3:4). For LXY30-KLVFFK(NH_2)-Beads and LXY30-KAAGGK(NH_2)-Beads, pheophorbride a (*Pa*) as a hydrophobic unit was linked. For LLP2A-KLVFFK(NH_2)-Beads and LLP2A-KAAGGK(NH_2)-Beads, succinic anhydride and resiquimod (*R848*) were successively conjugated. Then these peptides were cut off from the beads for 3 h using the solution with 82.5% TFA, 5% DI water, 5% phenol, 5% Thioanisole reagentplus, 2.5% Thiisopropylsilane. Cold ether was used to precipitate these peptide products. LXY30 ligands in LXY30-KLVFFK(*Pa*) and LXY30-KAAGGK(*Pa*) peptides were crosslinked *via* CLEAR-OX. In addition, 3-Methoxy-1-propanol was used to block the carboxyl group in LLP2A ligands *via* ester bond. After cold ether precipitation, *pro*LLP2A-KLVFFK(*R848*) and *pro*LLP2A-KAAGGK(*R848*) peptides were eventually acquired. Finally, these peptides were purified by High Performance Liquid Chromatography (HPLC, water 2998, US). The molecular weight of TPMs were confirmed by matrix-assisted laser desorption ionization time-off light mass spectrometry (MALDITOF mass spectra, Bruker Daltonics).

Self-assembly preparation and characterization of nanoparticles. TPMs were dissolved in DMSO to form a solution. Peptide solutions (LXY30-KLVFFK(*Pa*):*pro*LLP2A-KLVFFK(*R848*) is 1:1; 5 + 5 μL) was further diluted with DMSO (990, 790, 590, 390, 190, 90, 10 and 0 μL) and mixed with deionized water (0, 200, 400, 600, 800, 900, 980 and 990 μL). The ultraviolet-visible absorption (UV-vis) and fluorescence spectra (*ex*: 405 nm, UV-1800, Shimadzu and RF6000, Shimadzu) of solutions with varying water content were measured to validate the formation of nano-immuno-engager nanoparticles (NIE-NPs). The excitation and emission bandwidths were both set as 5.0 nm, and the data interval was 1 nm.

Fresh NIE-NPs, CNIE-NPs, NPs_{TPM1} and NPs_{TPM2} (99% water content, 20 μM) were used for measurement as an initial state. The morphology transformation of NIE-NPs to NIE-NFs was performed by the addition of $\alpha_3\beta_1$ integrin receptor protein or esterase plus $\alpha_4\beta_1$ integrin receptor protein (Sigma-Aldrich) and cultured for several hours at 37 °C. The NIE-NPs and NIE-NFs solution were used for size/zeta potential (DLS, Nano ZS), TEM measurement (CM-120 TEM, Phillips), circular dichroism (JASCO) and fluorescence spectra. TEM samples were dyed with uranyl acetate. Pyrene molecules were employed as an indicator to determine the CAC of nanoparticles, by comparing the fluorescence of their third and first emissive peaks. First, NIE-NPs or NIE-NFs was diluted to different concentrations (0.01, 0.05, 0.1, 0.5, 1, 5, 10, 20, 30 and 50 μM), then 999 μL of NIE-NPs or NIE-NFs of each dilution was incubated with 1 μL of pyrene acetone solution (0.1 mM) at 37 °C for 2 h. The fluorescence

spectra of pyrene (excitation, 335 nm) in different NIE-NPs or NIE-NFs dilutions were recorded. The fluorescence intensity ratio (I_3/I_1) of the third and first emissive peaks was measured for CAC calculation. Stability of NIE-NPs in the presence of human plasma and protease. The stability of NIE-NPs was studied in 10 % (v/v) plasma from healthy human volunteers and protease solution. The mixture was incubated at physiological body temperature (37 °C) followed by size measurements at predetermined time intervals up to 168 h.

Accumulated drug release of R848 from NIE-NFs was triggered by acidic pH 6.5 and porcine liver esterase (100 U mL^{-1}). Considering the experimental design (R848 was released from NIE-NFs in the tumor microenvironment), we firstly prepared NIE-NPs solution (100 μM), and then transformed into NIE-NFs after the interaction with $\alpha_3\beta_1$ integrin soluble protein. Then pH value in NIE-NFs solution was set up to 6.5 and porcine liver esterase was added into NIE-NFs solution. The mixture was incubated at 37 °C. At timed intervals, 20 μL solution was taken out and added into 80 μL DMSO for HPLC. Based on the pre-established calibration curves, free R848 concentration was calculated according to the integral area of free R848. Each value was reported as the means of the triplicate samples.

Cell line. The 4T1 breast cancer cell, Lewis lung cancer cell, MCF-7 breast cancer cell, A549 lung cancer cell and normal human peripheral blood mononuclear cell (PBMC, ATCC: PCS-800-011) were purchased from the American Type Culture Collection. Murine CD8 T cells were isolated from mouse spleen. Cell line authentication was performed by short tandem repeat DNA profiling. The cell line has been tested for mycoplasma contamination routinely. Cancer cell culture was performed in a cell incubator (5% carbon dioxide and 10% humidity. Temperature is 37 °C). These cancer cells were cultured in RPMI-1640 medium with 10% FBS and antibiotics containing penicillin and streptomycin.

***In vitro* cytotoxic assay.** 4T1 cells were used to evaluate the cytotoxicity of NIE-NPs and CNIE-NPs. Cells were seeded in 96-well plates (a density of 6000 cells per well, n = 3), cultured with RPMI-1640 and supplemented with 10% FBS and 1% penicillin at 37 °C in a humidified environment containing 5% CO_2. A 1% DMSO solution was diluted by RPMI-1640 (0.5, 1, 5, 10, 20 and 50 μM) and then added to each well for incubation with cells. After 48 h of incubation, MTS reagent was added into each well. The relative cell viabilities were measured by Micro-plate reader (SpectraMax M3, USA). Percentage of cell viability represented drug effect, and 100% means all cells survived. Cell viability was calculated using the following equation: Cell viability (%) =(OD490nm of treatment/OD490nm of blank control) × 100%.

CLSM and SEM validation of structural transformation on living cell surface. 4T1 cells were cultured in glass-bottom dishes for 12 h until all cells were completely attached. NIE-NPs and CNIE-NPs (50 μM) were incubated with cells in RPMI-1640 at 37 °C for different time, respectively. For confocal laser scanning microscopy imaging

(CLSM 800, ZEISS), specimens were fixed with glutaraldehyde (4%) for 10 min, washed with PBS three times, and examined with either a 63× immersion objective lens and a 405-nm laser. For SEM (Phillips XL30 TMP, FEI), cells were fixed with glutaraldehyde (4%) overnight and then coated with gold for 2 min. For stability of nanofibril on the cell surface experiment, 4T1 cells were firstly incubated with different NPs for 6 h, and the extra free NPs were removed. Then the cells continued to be incubated in the fresh medium without NPs for another 18 h. After that, specimens were fixed with glutaraldehyde (4%) for 10 min and washed with PBS three times for CLSM imaging. To simulate the processes of initial fibrillar transformation of NIE-NPs on the 4T1 cells surface followed by T cell binding and retention, NIE-NPs was first incubated with 4T1 cells for 6 h, unbound NIE-NPs were then washed off, followed by addition of fresh medium containing esterase but without NIE-NPs. After 1 h of incubation, activated murine CD8 T cells were added and incubated with 4T1 cells for 2 or 4 h. After that, unbound CD8 T cells were gently removed prior to CLSM imaging. CD8 T cells could be replaced by murine CD8 T cells. Similar experiment was performed using human CD8 T cells and human cancer cells (MCF-7 breast cancer cell and A549 lung cell).

The phenotype reeducation of tumor-associated macrophages. The bone marrow derived macrophage (BMDM) was firstly isolated, cultured and differentiated. In brief, femur and tibia bones from 6-8-week-old C57BL/6J mice were isolated, cleaned with PBS and cut open on both ends. Then a 21G needle and 10 ml syringe was used to flush out bone marrow into cold PBS plus 2% heat inactivated fetal bovine serum (FBS) (3-5 mL/mouse) and the marrow was allowed to pass through the needle 4-6 times to dissociate the cells. The bone marrow solution was then filtered through a 100 μm cell strainer to remove cell clumps, bone, hair and other cells/tissues. The collected cells mixture was mixed with 3 volume of RBC lysis buffer, and incubated on ice for 10min to remove red blood cells, followed by washing with PBS. The bone marrow cells were then cultured in BMDM growth medium (Iscove's Modified Dulbecco's Medium (IMDM) + 10% FBS + 10 ng/ml M-CSF) for 7 days to obtain mature macrophage cells. On day 7, for immune-suppressive M2-polarized phenotype activation, IMDM containing 10% FBS and 10 ng/mL IL-4 was added for 24 h; for anti-tumorigenic M1-polarized phenotype activation, R848 or NIE-NFs plus esterase, different incubation times were used.

Animal model. All animal experiments were performed in accordance with protocols No.19724, which was approved by the Animal Use and Care Administrative Advisory Committee at the University of California, Davis. The jugular vein of male Sprague-Dawley rats was cannulated, and a catheter was implanted for intravenous injection and blood collection (Harland, Indianapolis, IN, USA). NIE-NPs and CNIE-NPs (total 13 mg/kg) were i.v. administrated into rat (n = 3). Whole blood samples (~100 μL) were collected via jugular vein catheter before dosing and at predetermined time points post-injection. The whole blood samples were then centrifuged for serum collection, then the blood serum was diluted with DMSO (20 μL serum was added to 80 μL DMSO) for fluorescence measurements. The concentrations were measured by testing the fluorescence of *Pa* (*Ex* = 405 nm and *Em* = 675

nm). The values were calculated by molar concentration first, then exchanged to µg per mL. Female Balb/c mice were 6-8 weeks of age (weight 22 ± 2 g), which were purchased from Envigo. 4T1 cells (5×106 cells per mouse) were inoculated into the left mammary fat pad of each female Balb/c mice. After around 10 days, NIE-NPs and CNIE-NPs (total 13 mg/kg) were injected via the tail vein and *ex vivo* images of tumor, heart, liver, spleen, lung, kidney, intestine, muscle, skin were collected at 10, 24, 48, 72, 120 and 168 h post injection. The images were obtained by *in vivo* fluorescence imaging system (Carestream In-Vivo Imaging System FXPRO, USA). Tumors were excised and fixed with glutaraldehyde (4%) at 72 h post injection of NPs for TEM imaging.

Flow cytometry. To elucidate the mechanism of immunotherapeutic effects induced by bispecific NIE, the profile of tumor-infiltration immune cells was thoroughly evaluated in tumor tissues after treatment *via* flow cytometry analysis (CD45+, T cells, Macrophages, Dendritic cells, Neutrophiles, B cells and Monocytes, NK cells and Eosinophil). TILs were isolated by first digesting tumor tissue with collagenase type IV (2.5 mg/ml, Gibco) for one hour and the content was harvested after passing through 70 µm filter. Cells were stained with the respective fluorochrome conjugated monoclonal antibodies to the cell surface markers as follows: anti-CD3 (145-2C11), anti-CD8 (53-6.7), anti-CD45 (30-F11), anti-CD4 (RM4-5), anti-Foxp3 (NRRF30). The fluorescence stained cells were analyzed with a LSRFortessa (BD) and FlowJo software v.10 (TreeStar). Gating was performed based on the justification of first gate, exclusion of doublets by SSC-W and SSC-H, exclusion of dead cells by selection of Zombie aqua (BioLegend)/CD45+. CD3+ T cells, CD4+ T cells, CD8+ T cells, T_{reg} from TIL suspensions were analyzed using FlowJo software v.10. The results were based on the percentage of positively stained cells relative to upper gate cell number.

***In vivo* therapeutic effect.** Balb/c mice with 4T1 cells (5×10^6 cells per mouse) tumor inoculated into the left mammary fat pad were used in our experiments. The mice were randomly divided into six groups at 10 days posttumor inoculation. Each of them treated with different regimen every 48 h *via* i.v. administration, and the total treatment was 8 injections (13 mg/kg each dose). During the process of the treatment (21 days), the tumor volumes and body weight were measured every three days. The survival data was recorded till all mice were dead. For the synergistic treatment between different regimen and anti-PD-1, Balb/c mice with 4T1 tumor were randomly divided into four groups at 10 days post-tumor inoculation. Each of them treated with different regimen on day 1 *via* i.v. administration, and then anti-PD-1 was given *via* i.p. injection. A total of five such cycles are performed. During the process of the treatment (21 days), the tumor volumes were measured every three days. The survival data was recorded till 90 days. For re-challenge tumor experiment, 4T1 cells (5×10^6 cells per mouse) were re-inoculated into the mice previously treated with NIE-NPs (regimen 6) plus anti-PD-1 Ab (200 µg each mouse) on day 90. Same age naïve mice were used as a control group. During the process of the treatment (30 days), the tumor volumes were measured every three days. The survival data was recorded till 60 days. Tumor tissues were collected for

Haematoxylin and eosin (H&E), Ki-67, immunohistochemistry (IHC) staining, qPCR analysis and flow cytometric analysis images.

Statistical analysis.

Data are presented as the mean ± standard deviation (SD). The comparison between groups was analyzed with the student's *t*-test (two-tailed). Statistical significance was calculated using one-way ANOVA followed by Tukey's post hoc analysis; $*P < 0.05$, $**P < 0.01$, $***P < 0.001$, $****P < 0.0001$.

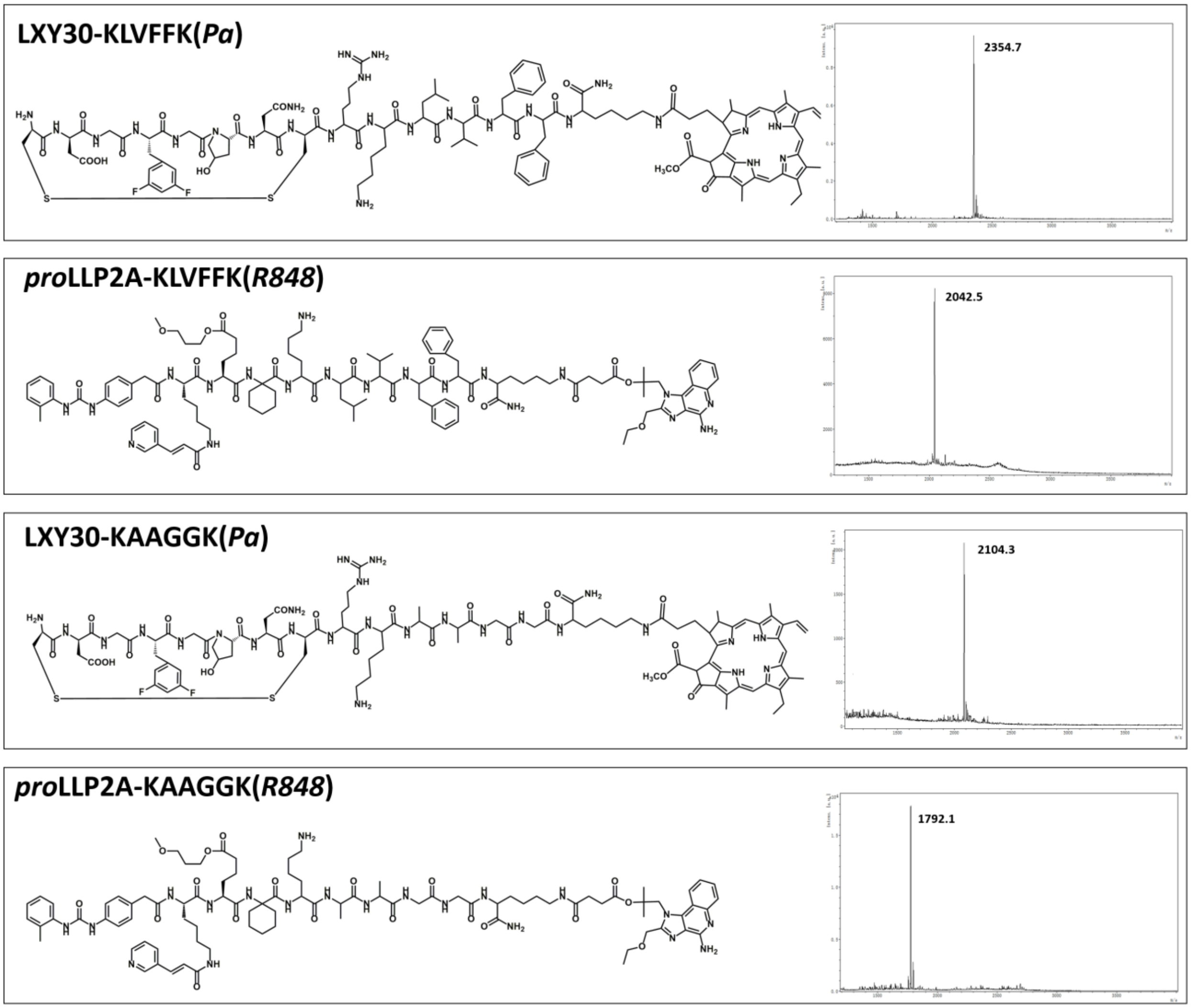

Figure S1. Chemical structure and mass spectra *via* MALDI-TOF of transformable peptide monomer: TPM1 LXY30-KLVFFK(*Pa*), TPM2 *pro*LLP2A-KLVFFK(*R848*), CTPM3 LXY30-KAAGGK(*Pa*), CTPM4 *pro*LLP2A-KAAGGK(*R848*).

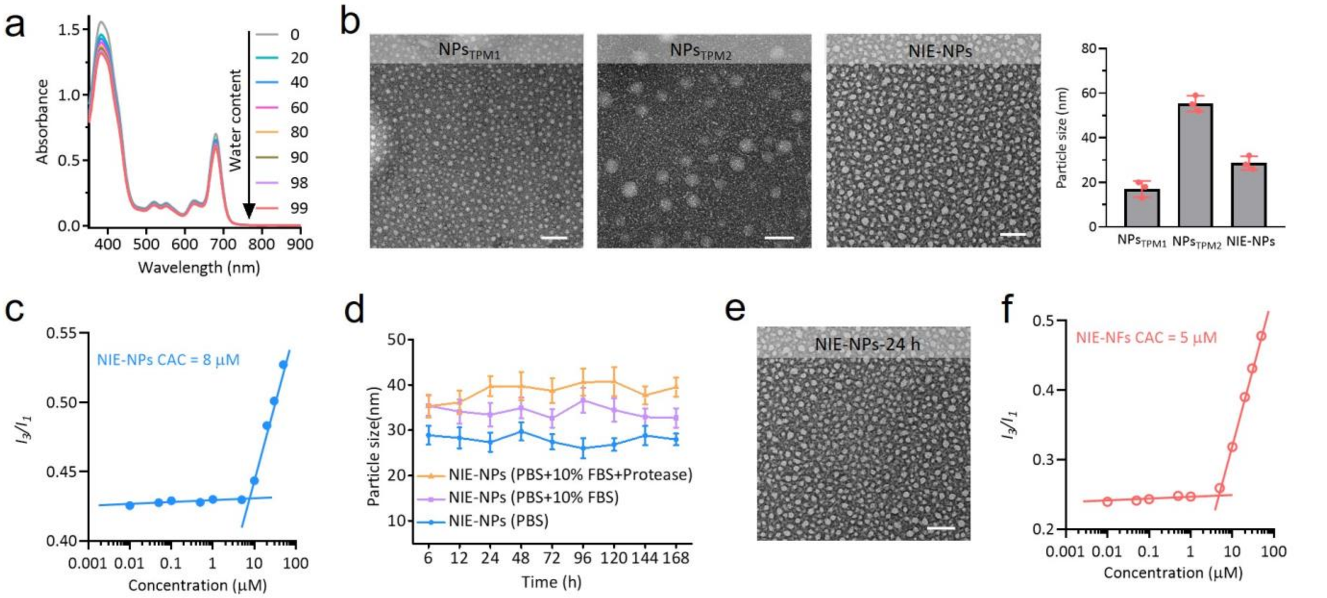

Figure S2. a, Changes in UV imaging of DMSO solution of TPM1 and TPM2 at a 1:1 ratio following the gradual addition of water (from 0 to 99%) forming NIE-NPs. **b**, TEM images and size distribution of NPs_{TPM1}, NPs_{TPM1} and NIE-NPs at the H_2O and DMSO ratio of 99:1. **c**, The critical aggregation concentration (CAC) of NIE-NPs was measured by using pyrene as a probe. **d**, Nanoparticle stability of NIE-NPs in serum and protease (PBS solution of pH 7.4 with/without 10% FBS and protease) at 37 ^{o}C was measured by dynamic light scattering. Data are presented as the mean ± s.d., $n = 3$ independent experiments. **e**, TEM images of NIE-NPs after 24 h in PBS solution. **f**, The CAC of NIE-NFs was measured by using pyrene as a probe. The scale bar in all TEM images is 100 nm. The concentration of NIE-NPs used in **b**, **d**, **e** was 20 μM.

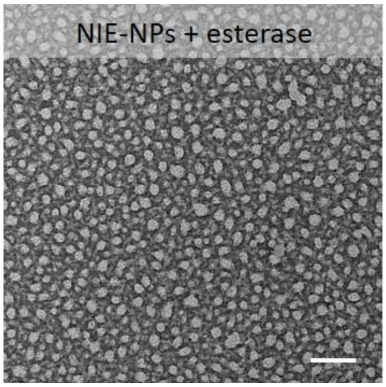

Figure S3. TEM images of NIE-NPs interaction with esterase for 24 h. The concentration of NIE-NPs used in the experiment was 20 μM. The scale bars are 100 nm.

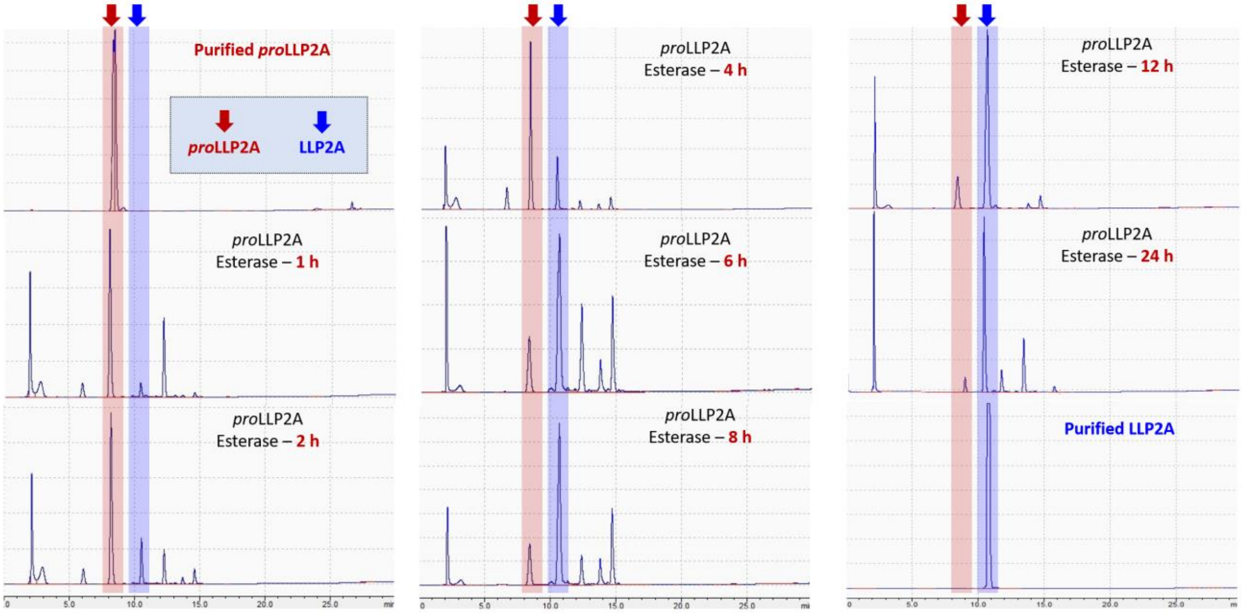

Figure S4. HPLC monitoring of conversion process of *pro*LLP2A ligand to LLP2A ligand with esterase (porcine liver esterase: 100 U/mL) at 37 °C (pH = 7.4) and the control purified LLP2A ligand. The peak of LLP2A was found at 1 h post-interaction of *pro*LLP2A ligand with esterase. The ester bonds were broken very quickly. After 6 h, the peak of LLP2A ligand was higher than that of *pro*LLP2A ligand. By 24 h, the vast majority of *pro*LLP2A ligands had been converted to LLP2A ligands. This shows that esterase is very efficient at breaking ester bonds to convert LLP2A ligand.

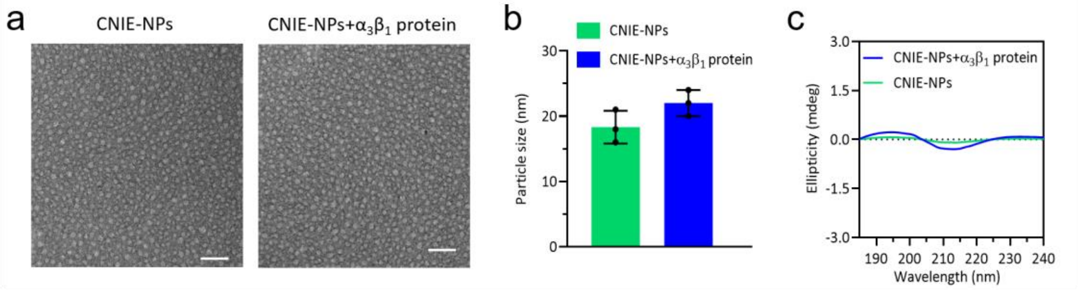

Figure S5. a,b, TEM images (**a**) and particle size (**b**) of initial CNIE-NPs and CNIE-NPs interaction with $\alpha_3\beta_1$ integrin protein for 24 h. The molar ratio of $\alpha_3\beta_1$ integrin protein/peptide ligand was approximately 1:1000. The scale bar is 100 nm. The concentration of CNIE-NPs used in the experiment was 20 μM. **c**, Circular dichroism spectra of initial CNIE-NPs and CNIE-NPs interaction with $\alpha_3\beta_1$ integrin protei.

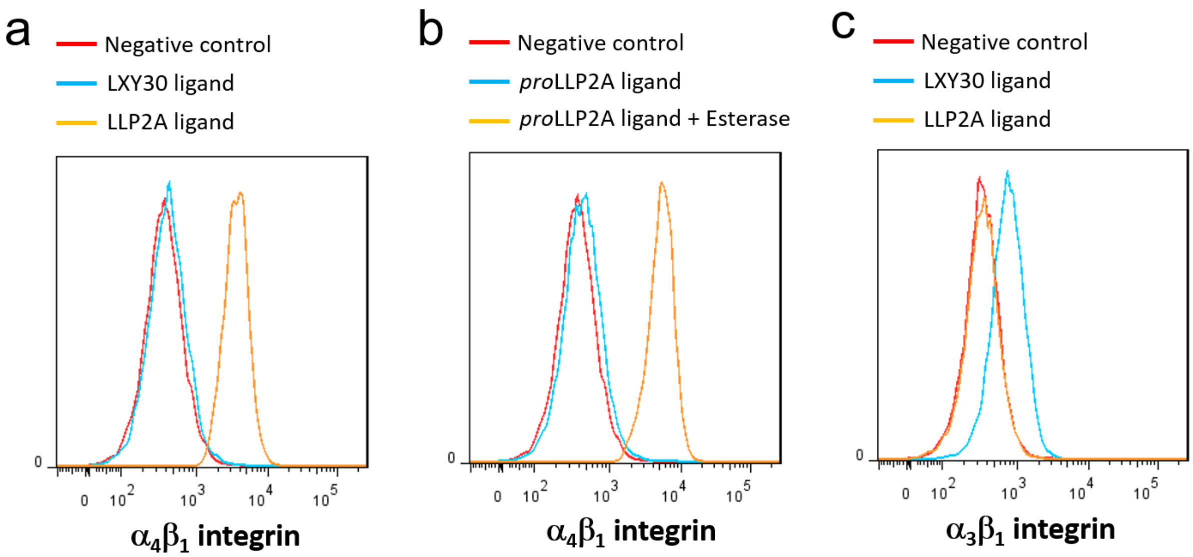

Figure S6. Biotinylated LXY30, LLP2A, *pro*LLP2A and *pro*LLP2A/esterase incubated with $\alpha_3\beta_1$ or $\alpha_4\beta_1$ transfected K562 cells. 3*10^5 cells incubated with 1 µM biotinylated ligand peptides in binding buffer (1x PBS, 10% FBS, 1 mM Mn^{2+}) for 30 min on ice, after washing with PBS followed by incubation with 1:500 streptavidin-PE (1 mg/mL) for 30 min, and then flow cytometry analysis.

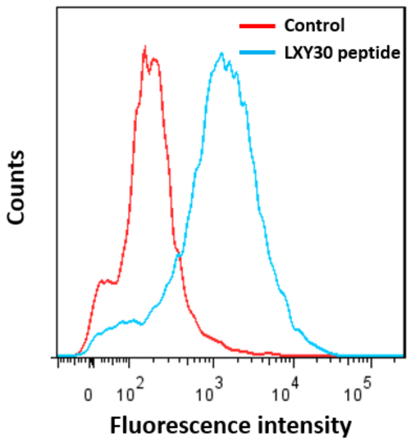

Figure S7. Biotinylated LXY30 peptide (blue curve) and negative control (red curve) incubation with 4T1 cells were analyzed with flow cytometry. 3*10^5 cells incubated with 1 µM biotinylated LXY30 for 30 min on ice, after washing with PBS followed by incubation with 1:500 streptavidin-PE (1mg/mL) for 30min, and then flow cytometry analysis.

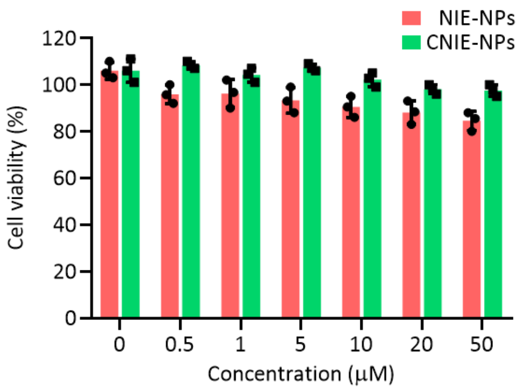

Figure S8. Viability of 4T1 cells after incubation with NIE-NPs and CNIE-NPs at different concentrations for 48 h. Data are presented as mean ± s.d., $n = 3$ independent experiments.

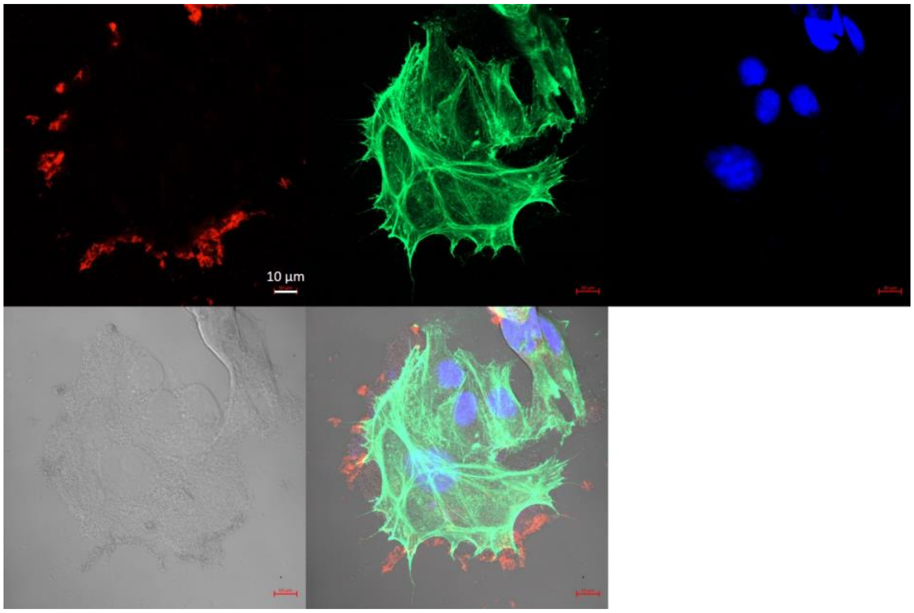

Figure S9. Fluorescent microscopy of 4T1 tumor cells in cell culture, 6 h after incubation with NIE-NPs (red fluorescence) which was found surrounding the tumor cells. Green fluorescence depicts actins stained by phalloidin; blue fluorescence depicts nuclei stained by DAPI. The concentration of NIE-NPs used was 50 μM.

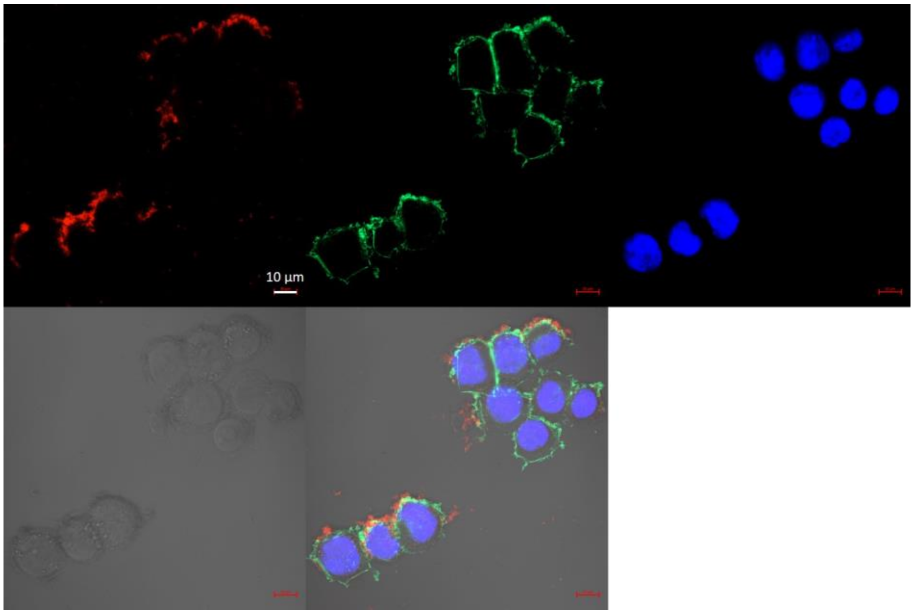

Figure S10. Fluorescent microscopy of 4T1 tumor cells in cell culture, 6 h after incubation with NIE-NPs (red fluorescence) which was found surrounding the tumor cells. Green fluorescence depicts cell membrane stained with DiO; blue fluorescence depicts nuclei stained by DAPI. The concentration of NIE-NPs was 50 µM.

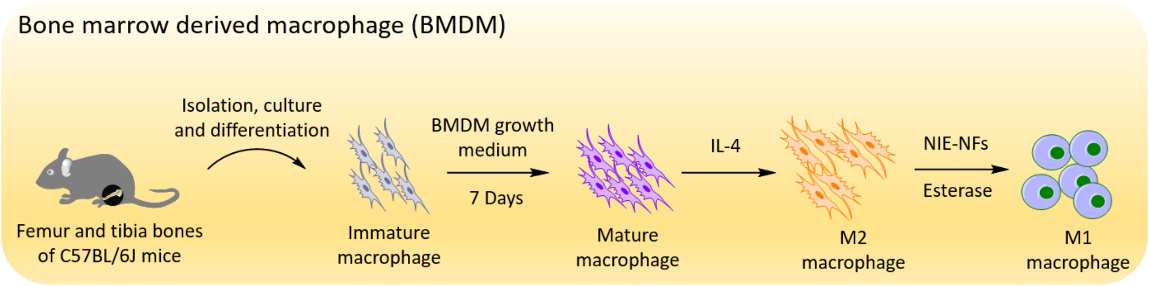

Figure S11. Schematic illustration of the inducing process of M2-like and M1-like tumor-associated macrophage under different conditions.

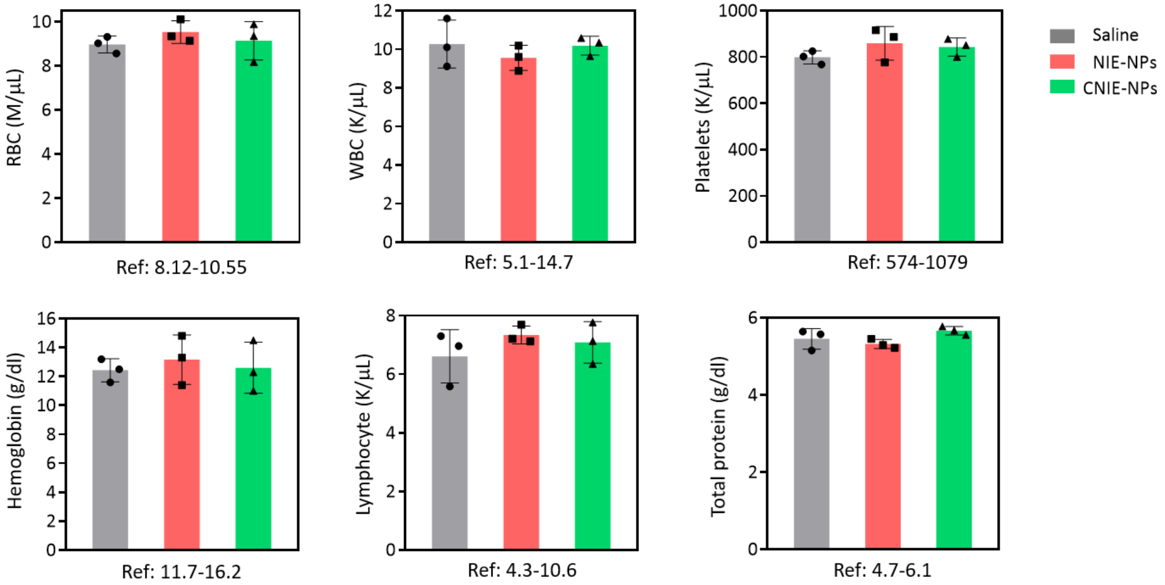

Figure S12. Blood test parameters in terms of red blood cells (RBC), white blood cells (WBC), platelets, hemoglobin, lymphocyte and total protein of healthy Balb/c mice, after 8 q.o.d. intravenous injections of NIE-NPs and CNIE-NPs (13 mg/kg per injection). Data are presented as the mean ± s.d., $n = 3$ independent experiments.

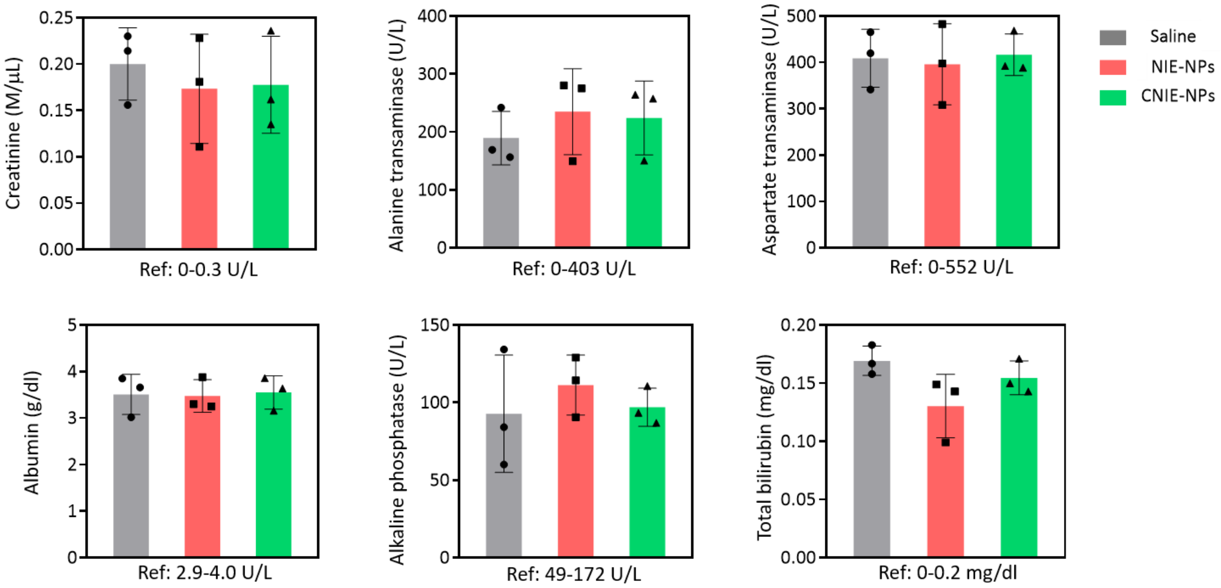

Figure S13. Blood test parameters in terms of liver function creatinine, alanine transaminase, aspartate transaminase, albumin, alkaline phosphatase, total bilirubin of healthy Balb/c mice after 8 q.o.d. intravenous injection of NIE-NPs and CNIE-NPs (13 mg/kg per injection). Data are presented as the mean ± s.d., $n = 3$ independent experiments.

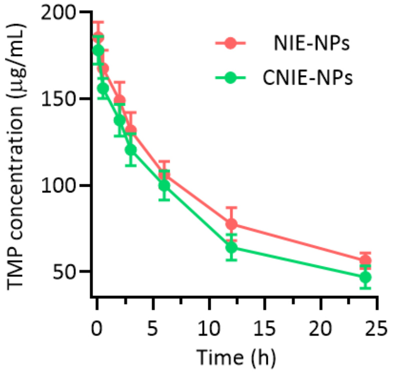

Group	C-max (μg/mL)	AUC	T-half (α)/h	T-half (β)/h
NIE-NPs	193.12	1969.572	2.866	23.1862
CNIE-NPs	186.85	1875.363	2.628	21.9657

Figure S14. *In vivo* blood pharmacokinetics and parameter of NIE-NPs and CNIE-NPs (Data are presented as the mean ± s.d., *n* = 3 independent experiments). The C-max, AUC and $T_{1/2}$ (hours) were calculated by Kinetica 5.0.

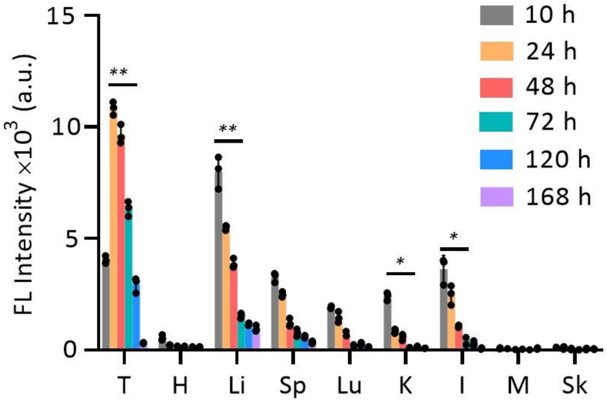

Figure S15. Time-dependent *ex vivo* fluorescence (FL) quantitative analysis of tumor tissues and major organs (heart (H), liver (Li), spleen (Sp), lung (Lu), kidney (K), intestine (I), muscle (M) and skin (Sk)) collected at 10, 24, 48, 72, 120 and 168 h post-injection of NIE-NPs. Data are presented as mean ± s.d., *n* = 3 independent experiments.

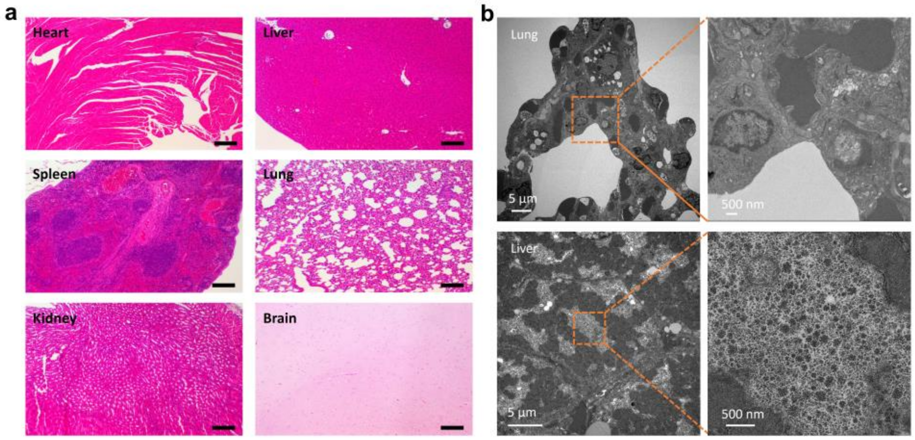

Figure S16. (a) H&E stain of major organs did not show any systemic toxicity caused by NIE-NPs 72 h after i.v. injection. Experiments were repeated three times. The scale bar is 200 µm. (b) TEM images of lung and liver excised 10 h after i.v. injection of NIE-NPs. The level of NIE-NPs used was 13 mg/kg.

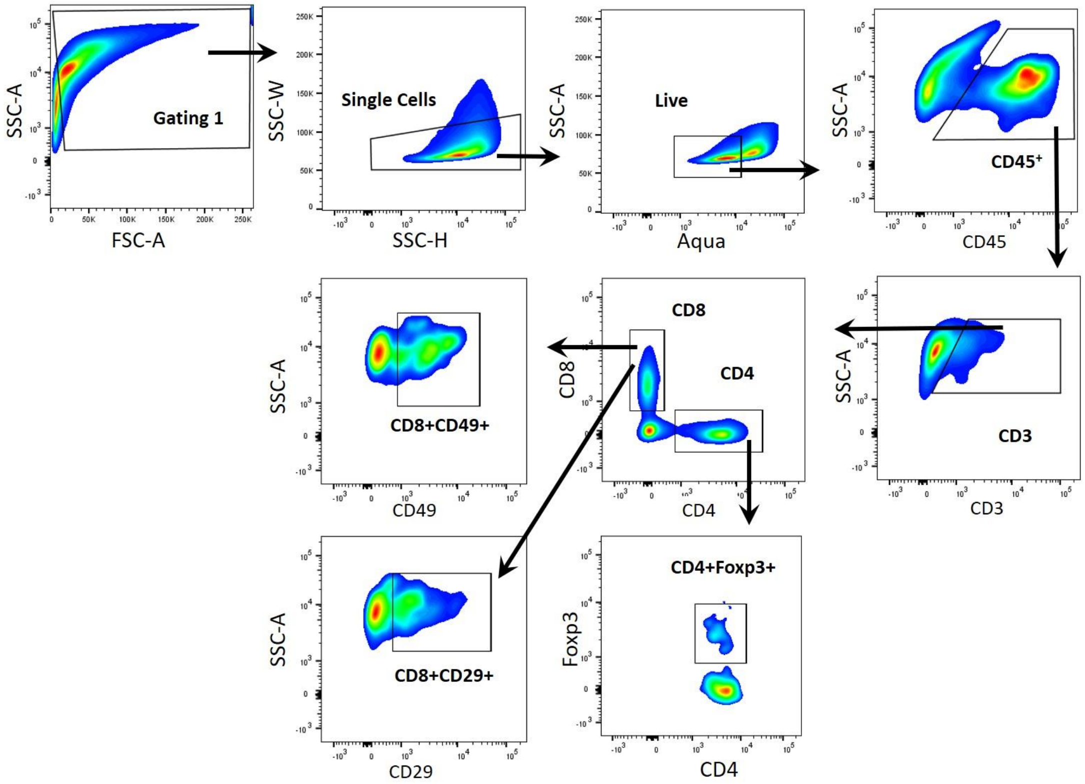

Figure S17. Gating strategies for flow cytometric analysis of CD8 and CD4 T cells, CD49 and CD29 in CD8 T cell and CD4 T cells within the 4T1 tumors tissue.

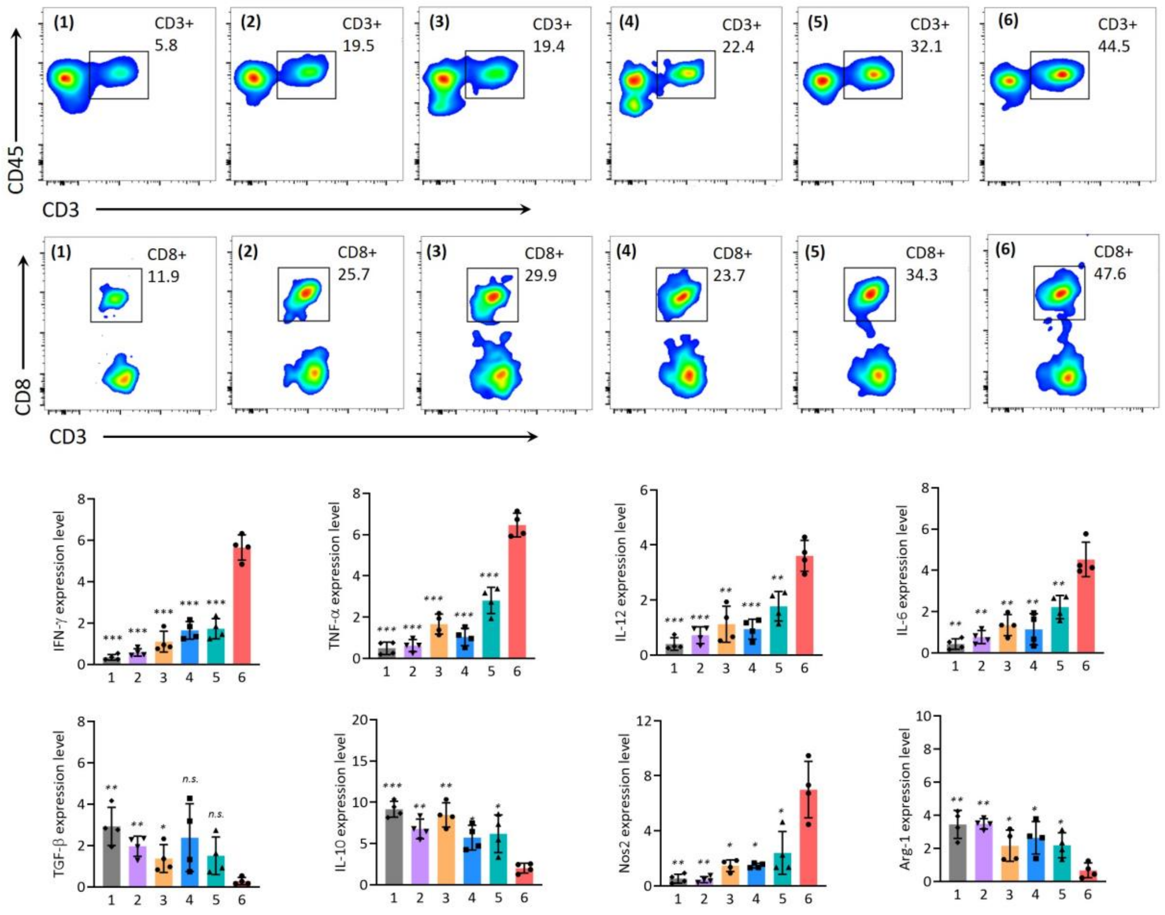

Figure S18. Representative flow cytometric analysis images of $CD45^{+}CD3^{+}$, $CD3^{+}CD8^{+}$ T cell within the excised 4T1 tumors from mice treated with: (1) Saline; (2) $(EK)_3$-KLVFFK(*Pa*)/$(EK)_3$-KLVFFK(*R848*) (micellar R848); (3) *pro*LLP2AKLVFFK(R848) (single monomer); (4) LXY30-KAAGGK(*Pa*)/*pro*LLP2A-KAAGGK(*R848*) (untransformable negative control CNIE-NPs); (5) LXY30-KLVFFK(*Pa*)/*pro*LLP2A-KLVFFK(*Pa*) (fibrillar transformation but absence of R848); (6) LXY30-KLVFFK(*Pa*)/*pro*LLP2A-KLVFFK(*R848*) (NIE-NPs). The expression levels (analyzed by qPCR) of IFN-γ, TNF-α, IL12, IL6, TGF-β, IL10, Nos2 and Arg-1 in 4T1 tumors excised from mice on day 21 (data were mean ± s.d.). *$*P < 0.05$, $**P < 0.01$, $***P < 0.001$, $****P < 0.0001$*.

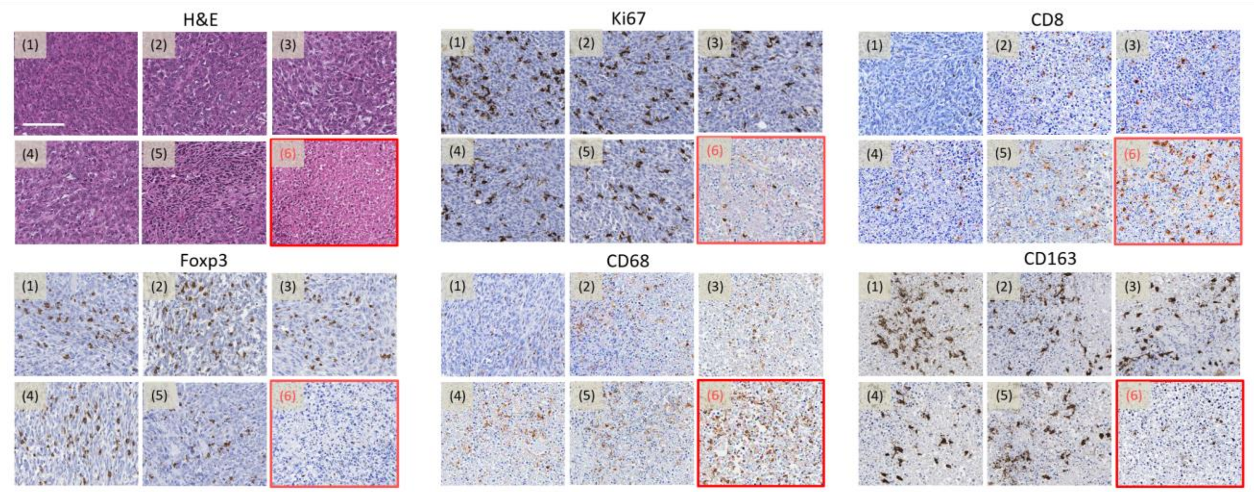

Figure S19. H&E and IHC images of excised tumors. Representative images are shown for the IHC staining of Ki67, T cells (CD8, Foxp3) and macrophage markers (CD68, CD163). Scale bar is 100 μm.

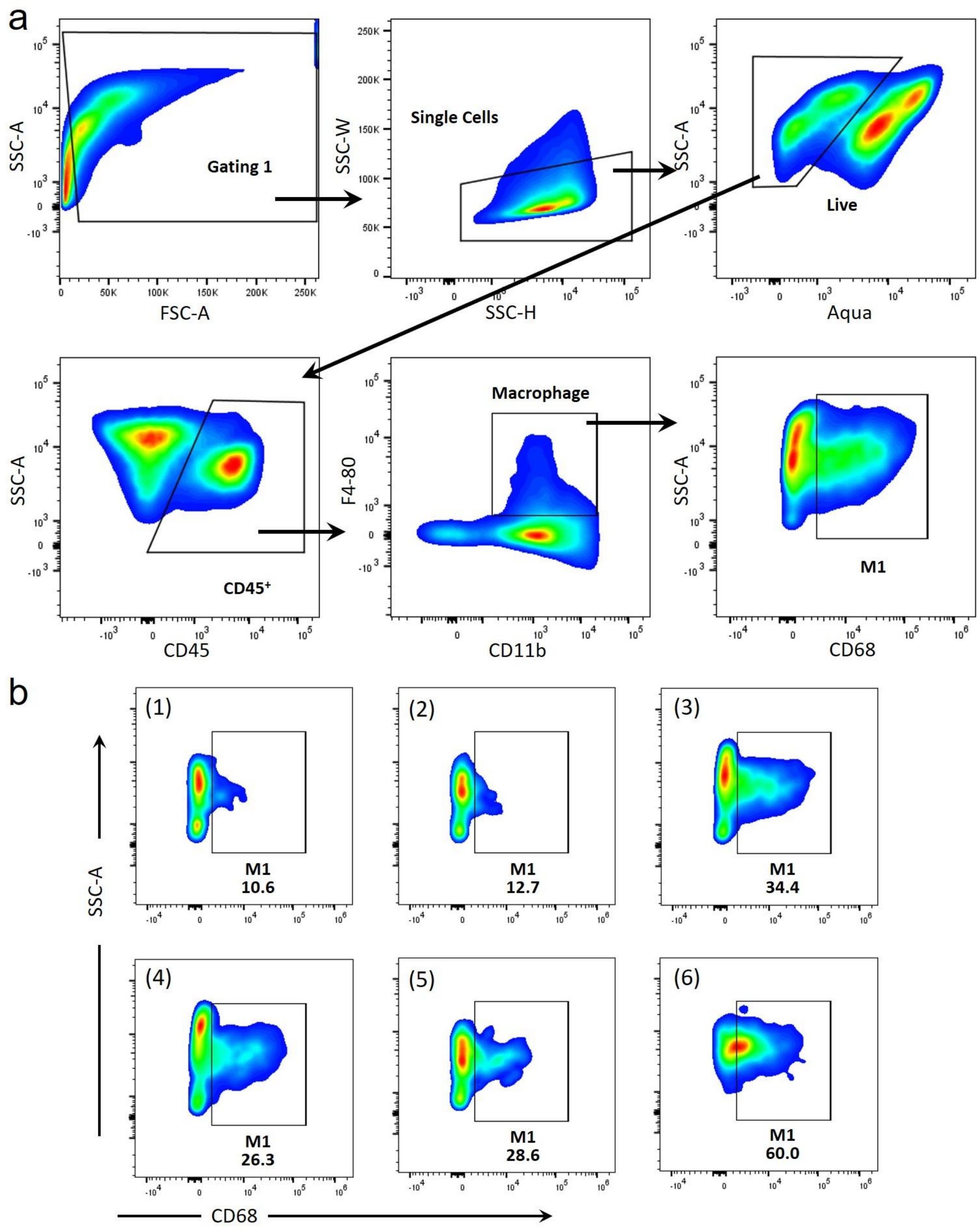

Figure S20. a, Gating strategies for flow cytometric analysis of M1-phenotype macrophage within the 4T1 tumors. **b,** Representative flow cytometric analysis images of M1-phenotype macrophage within the 4T1 tumors excised from treated mice on day 21.

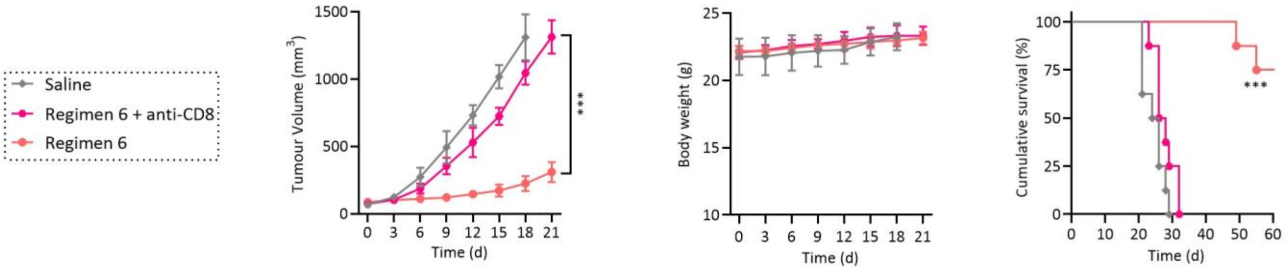

Figure S21. Observation of tumor inhibitory effect, weight change and cumulative survival of mice bearing orthotopic 4T1 tumor treated by regimen 6 plus anti-CD8 antibody (CD8 T cell depletion) and regimen 6 alone (n = 8 per group). Anti-CD8 depletion antibody (i.p. injection) was given at an initial dose of 200 μg 3 days before treatment and then given four doses on day 1, 5, 9 and 13. When tumor volume reached about 100 mm^3, NIE-NPs were injected via tail vein consecutively eight times q.o.d. (13 mg/kg each dose, every two days) and the mice were continuously observed for 21 days. Data are presented as mean ± s.d.
